# Supplementary figures and images for: Activation of transient receptor potential vanilloid 4 is involved in pressure overload-induced cardiac hypertrophy (part 2 of 2)
Source: eLife. 2022 Jun 22;11:e74519. doi: 10.7554/eLife.74519 (PMC9224988; doi:10.7554/eLife.74519)

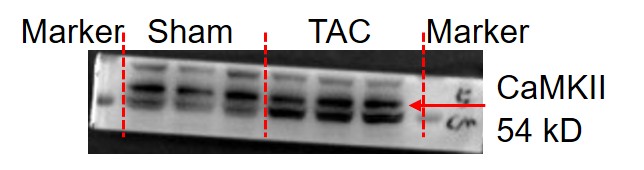

Supplement: Source data 1. [file elife-74519-data1.zip › Source data 1/Western blots/labelled/figure8G-labelled/WT-TAC2w-CaMKII-2-labelled.jpg]

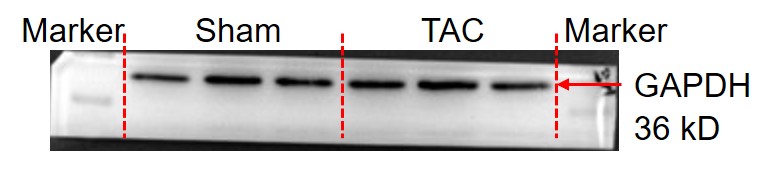

Supplement: Source data 1. [file elife-74519-data1.zip › Source data 1/Western blots/labelled/figure8G-labelled/WT-TAC2w-GAPDH-1-labelled.jpg]

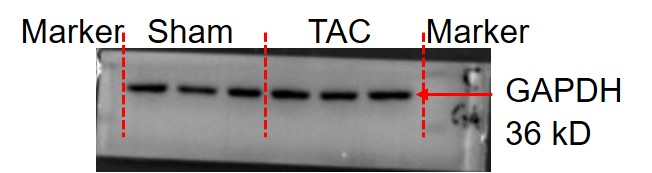

Supplement: Source data 1. [file elife-74519-data1.zip › Source data 1/Western blots/labelled/figure8G-labelled/WT-TAC2w-GAPDH-2-labelled.jpg]

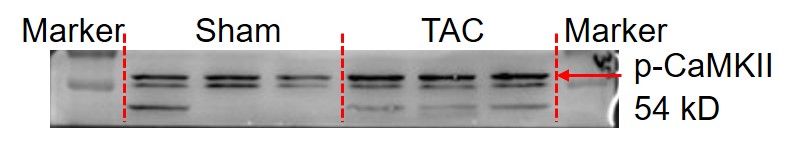

Supplement: Source data 1. [file elife-74519-data1.zip › Source data 1/Western blots/labelled/figure8G-labelled/WT-TAC2w-pCaMKII-1-labelled.jpg]

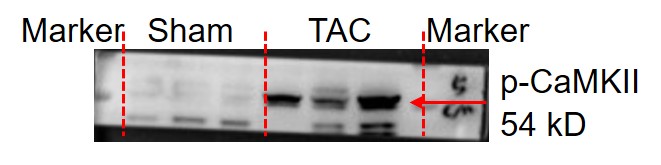

Supplement: Source data 1. [file elife-74519-data1.zip › Source data 1/Western blots/labelled/figure8G-labelled/WT-TAC2w-pCaMKII-2-labelled.jpg]

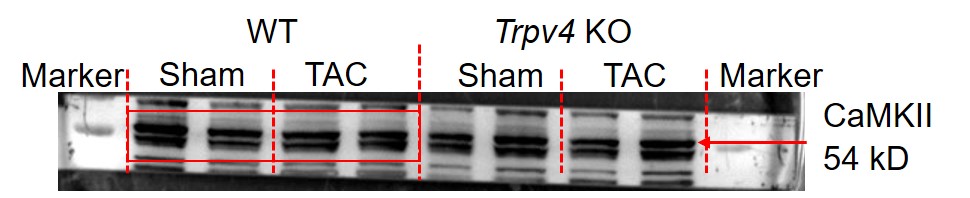

Supplement: Source data 1. [file elife-74519-data1.zip › Source data 1/Western blots/labelled/figure8G-labelled/WT-TAC4w-CaMKII-1-labelled.jpg]

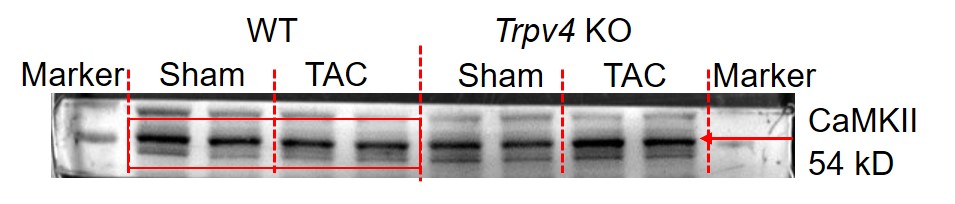

Supplement: Source data 1. [file elife-74519-data1.zip › Source data 1/Western blots/labelled/figure8G-labelled/WT-TAC4w-CaMKII-2-labelled.jpg]

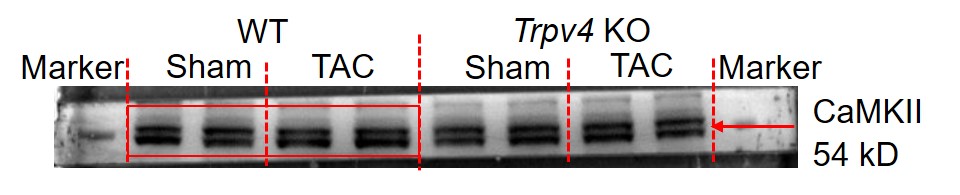

Supplement: Source data 1. [file elife-74519-data1.zip › Source data 1/Western blots/labelled/figure8G-labelled/WT-TAC4w-CaMKII-3-labelled.jpg]

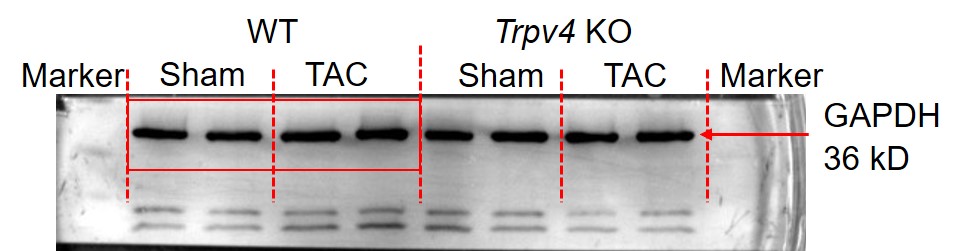

Supplement: Source data 1. [file elife-74519-data1.zip › Source data 1/Western blots/labelled/figure8G-labelled/WT-TAC4w-GAPDH-1-labelled.jpg]

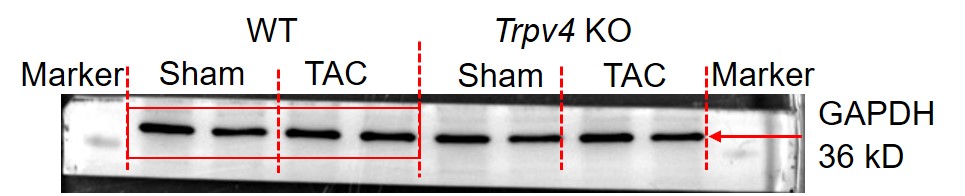

Supplement: Source data 1. [file elife-74519-data1.zip › Source data 1/Western blots/labelled/figure8G-labelled/WT-TAC4w-GAPDH-2-labelled.jpg]

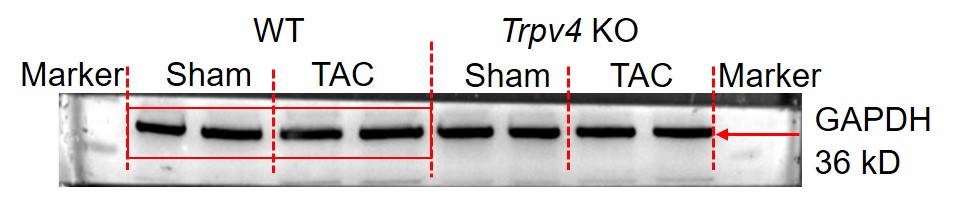

Supplement: Source data 1. [file elife-74519-data1.zip › Source data 1/Western blots/labelled/figure8G-labelled/WT-TAC4w-GAPDH-3-labelled.jpg]

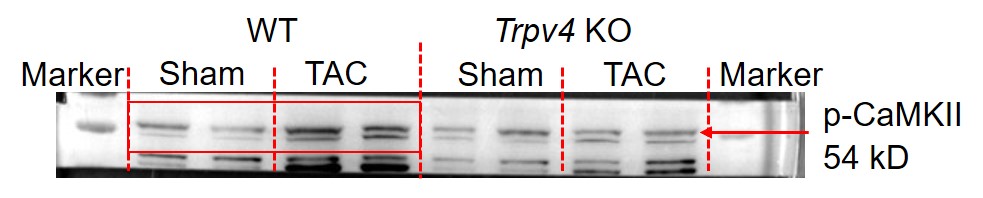

Supplement: Source data 1. [file elife-74519-data1.zip › Source data 1/Western blots/labelled/figure8G-labelled/WT-TAC4w-pCaMKII-1-labelled.jpg]

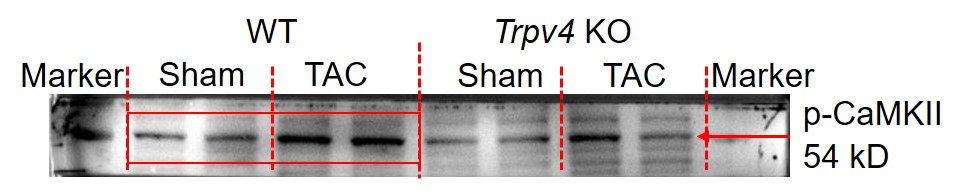

Supplement: Source data 1. [file elife-74519-data1.zip › Source data 1/Western blots/labelled/figure8G-labelled/WT-TAC4w-pCaMKII-2-labelled.jpg]

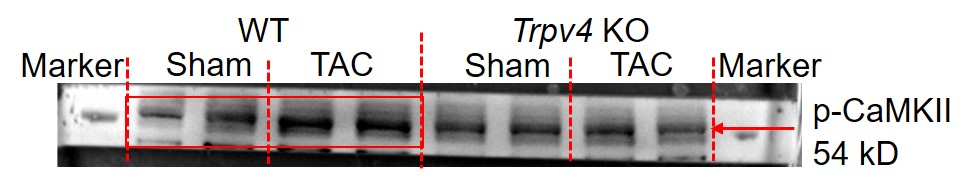

Supplement: Source data 1. [file elife-74519-data1.zip › Source data 1/Western blots/labelled/figure8G-labelled/WT-TAC4w-pCaMKII-3-labelled.jpg]

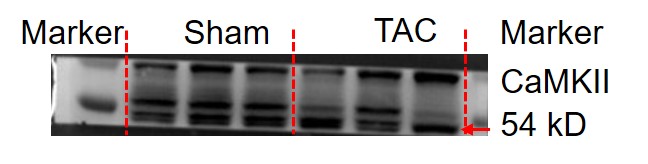

Supplement: Source data 1. [file elife-74519-data1.zip › Source data 1/Western blots/labelled/figure8I-labelled/TRPV4 KO-TAC1w-CaMKII-1-labelled.jpg]

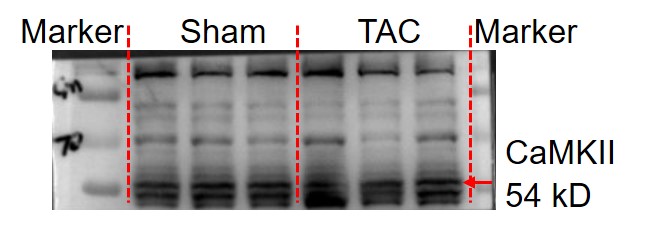

Supplement: Source data 1. [file elife-74519-data1.zip › Source data 1/Western blots/labelled/figure8I-labelled/TRPV4 KO-TAC1w-CaMKII-2-labelled.jpg]

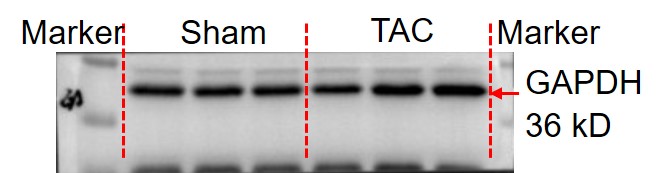

Supplement: Source data 1. [file elife-74519-data1.zip › Source data 1/Western blots/labelled/figure8I-labelled/TRPV4 KO-TAC1w-GAPDH-1-labelled.jpg]

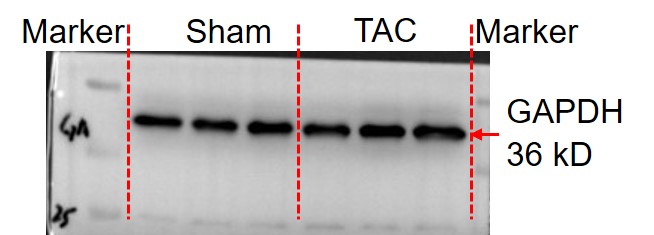

Supplement: Source data 1. [file elife-74519-data1.zip › Source data 1/Western blots/labelled/figure8I-labelled/TRPV4 KO-TAC1w-GAPDH-2-labelled.jpg]

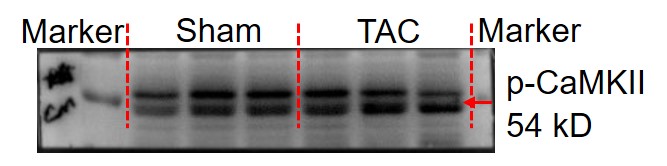

Supplement: Source data 1. [file elife-74519-data1.zip › Source data 1/Western blots/labelled/figure8I-labelled/TRPV4 KO-TAC1w-pCaMKII-1-labelled.jpg]

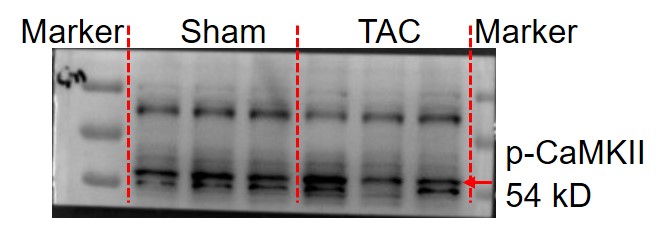

Supplement: Source data 1. [file elife-74519-data1.zip › Source data 1/Western blots/labelled/figure8I-labelled/TRPV4 KO-TAC1w-pCaMKII-2-labelled.jpg]

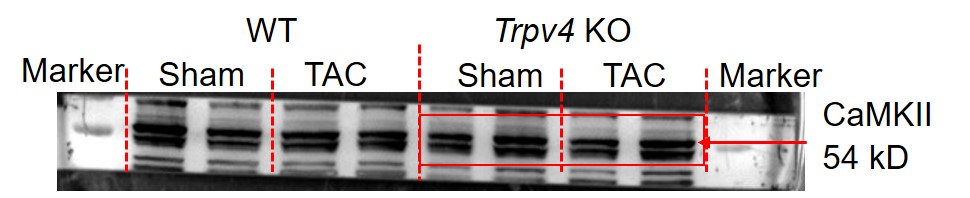

Supplement: Source data 1. [file elife-74519-data1.zip › Source data 1/Western blots/labelled/figure8I-labelled/Trpv4 KO-TAC4w-CaMKII-1-labelled.jpg]

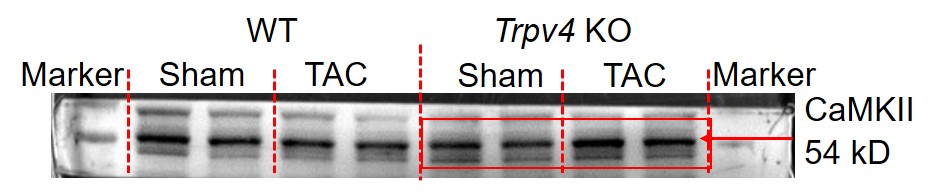

Supplement: Source data 1. [file elife-74519-data1.zip › Source data 1/Western blots/labelled/figure8I-labelled/Trpv4 KO-TAC4w-CaMKII-2-labelled.jpg]

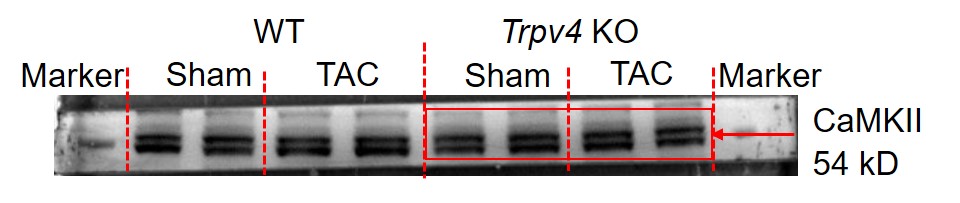

Supplement: Source data 1. [file elife-74519-data1.zip › Source data 1/Western blots/labelled/figure8I-labelled/Trpv4 KO-TAC4w-CaMKII-3-labelled.jpg]

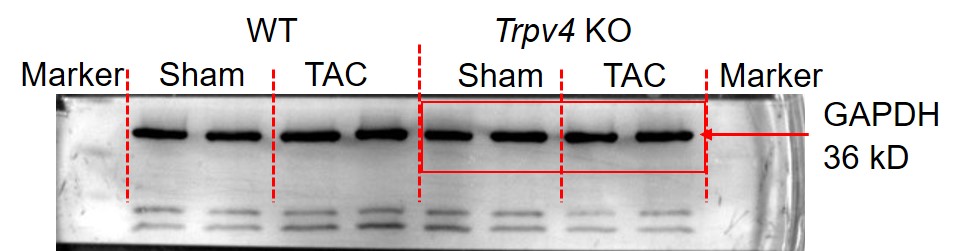

Supplement: Source data 1. [file elife-74519-data1.zip › Source data 1/Western blots/labelled/figure8I-labelled/Trpv4 KO-TAC4w-GAPDH-1-labelled.jpg]

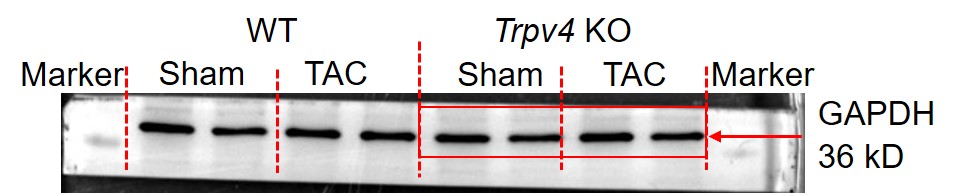

Supplement: Source data 1. [file elife-74519-data1.zip › Source data 1/Western blots/labelled/figure8I-labelled/Trpv4 KO-TAC4w-GAPDH-2-labelled.jpg]

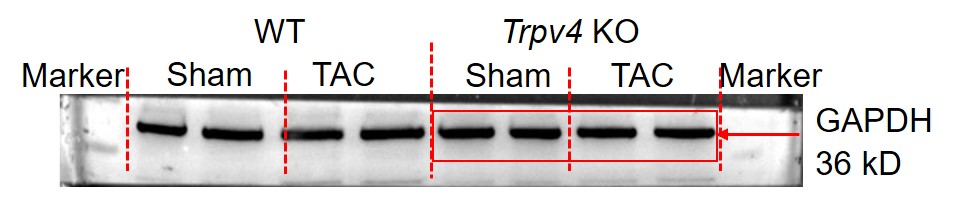

Supplement: Source data 1. [file elife-74519-data1.zip › Source data 1/Western blots/labelled/figure8I-labelled/Trpv4 KO-TAC4w-GAPDH-3-labelled.jpg]

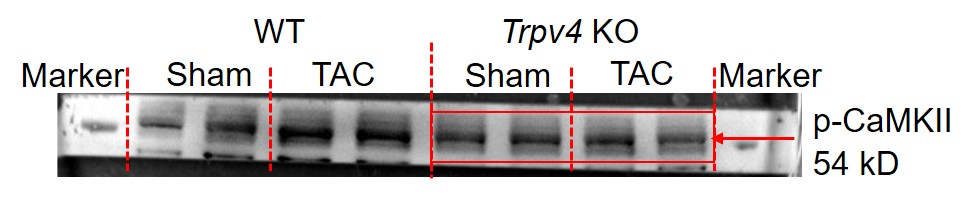

Supplement: Source data 1. [file elife-74519-data1.zip › Source data 1/Western blots/labelled/figure8I-labelled/Trpv4 KO-TAC4w-pCaMKII-1-labelled.jpg]

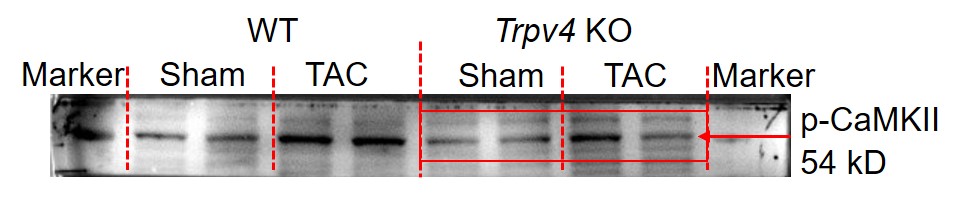

Supplement: Source data 1. [file elife-74519-data1.zip › Source data 1/Western blots/labelled/figure8I-labelled/Trpv4 KO-TAC4w-pCaMKII-2-labelled.jpg]

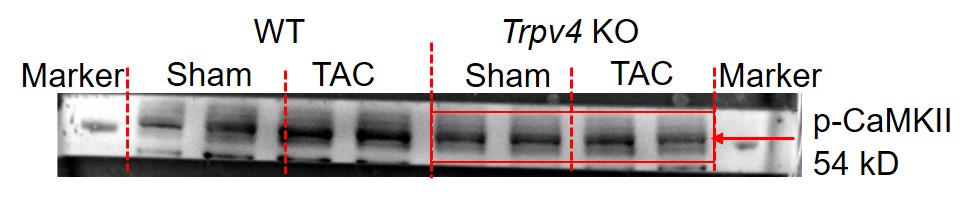

Supplement: Source data 1. [file elife-74519-data1.zip › Source data 1/Western blots/labelled/figure8I-labelled/Trpv4 KO-TAC4w-pCaMKII-3-labelled.jpg]

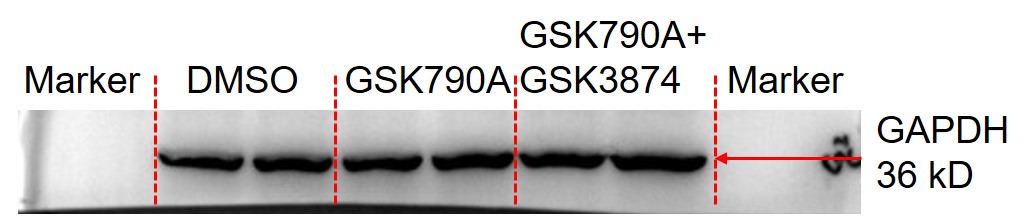

Supplement: Source data 1. [file elife-74519-data1.zip › Source data 1/Western blots/labelled/figure9A-labelled/figure9A-GAPDH-1-labelled.jpg]

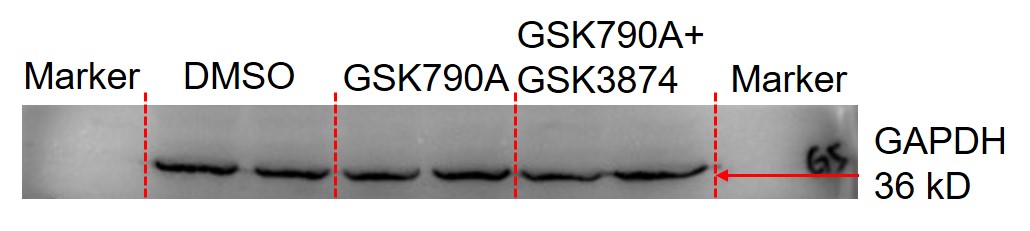

Supplement: Source data 1. [file elife-74519-data1.zip › Source data 1/Western blots/labelled/figure9A-labelled/figure9A-GAPDH-2-labelled.jpg]

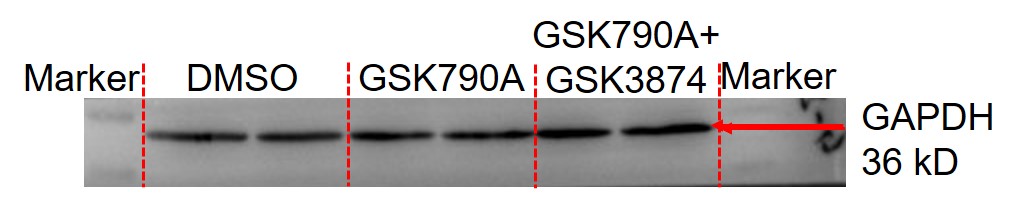

Supplement: Source data 1. [file elife-74519-data1.zip › Source data 1/Western blots/labelled/figure9A-labelled/figure9A-GAPDH-3-labelled.jpg]

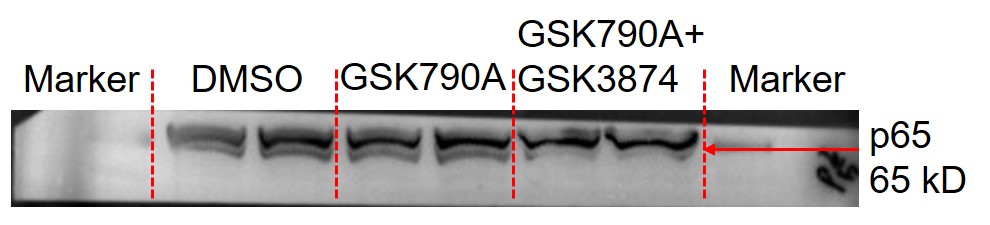

Supplement: Source data 1. [file elife-74519-data1.zip › Source data 1/Western blots/labelled/figure9A-labelled/figure9A-p65-1-labelled.jpg]

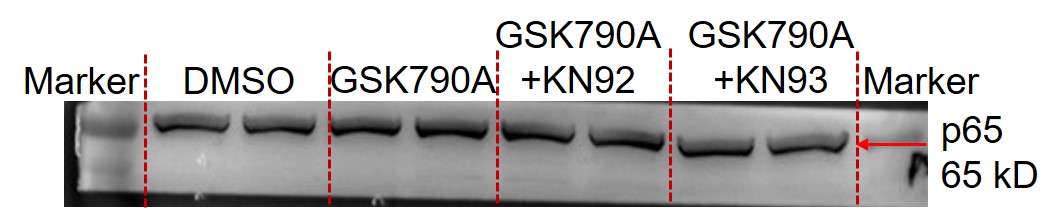

Supplement: Source data 1. [file elife-74519-data1.zip › Source data 1/Western blots/labelled/figure9A-labelled/figure9A-p65-2-labelled.jpg]

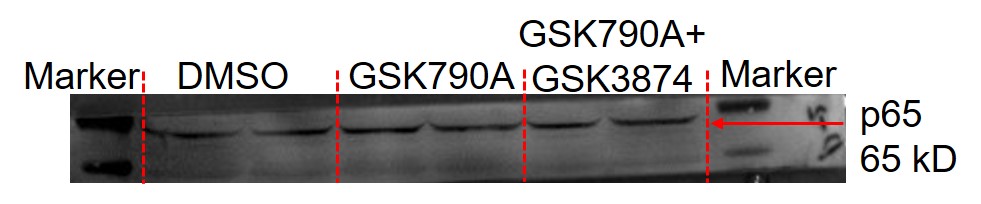

Supplement: Source data 1. [file elife-74519-data1.zip › Source data 1/Western blots/labelled/figure9A-labelled/figure9A-p65-3-labelled.jpg]

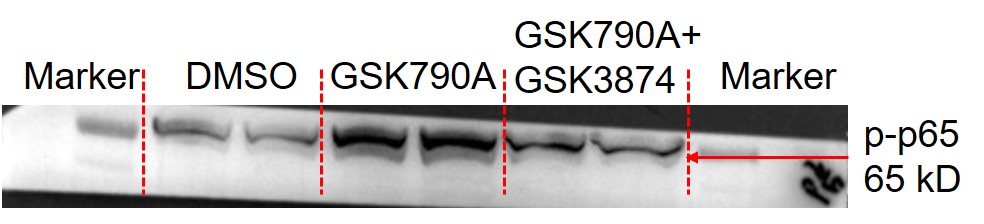

Supplement: Source data 1. [file elife-74519-data1.zip › Source data 1/Western blots/labelled/figure9A-labelled/figure9A-pp65-1-labelled.jpg]

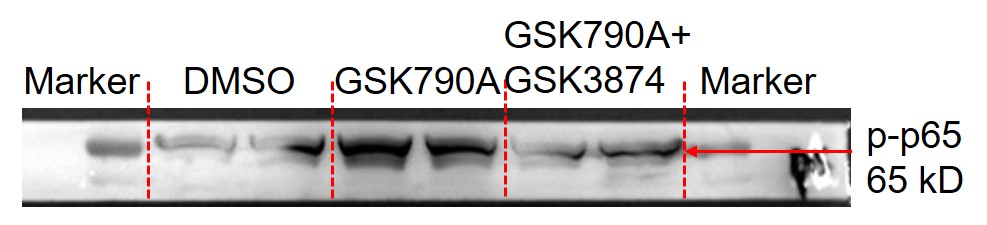

Supplement: Source data 1. [file elife-74519-data1.zip › Source data 1/Western blots/labelled/figure9A-labelled/figure9A-pp65-2-labelled.jpg]

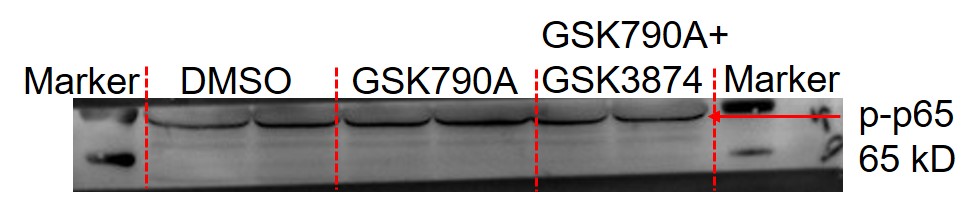

Supplement: Source data 1. [file elife-74519-data1.zip › Source data 1/Western blots/labelled/figure9A-labelled/figure9A-pp65-3-labelled.jpg]

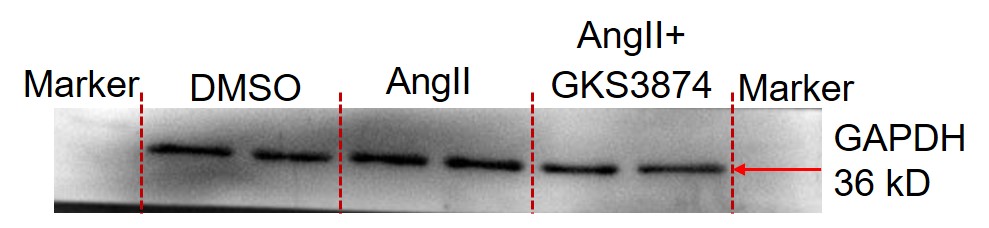

Supplement: Source data 1. [file elife-74519-data1.zip › Source data 1/Western blots/labelled/figure9C-labelled/figure9C-GAPDH-1-labelled.jpg]

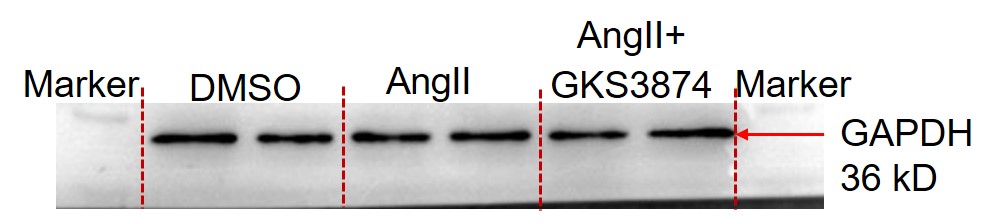

Supplement: Source data 1. [file elife-74519-data1.zip › Source data 1/Western blots/labelled/figure9C-labelled/figure9C-GAPDH-2-labelled.jpg]

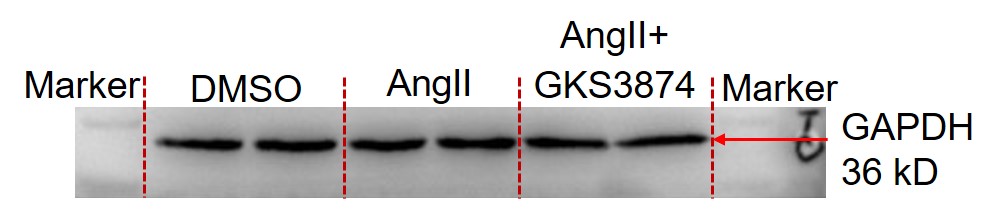

Supplement: Source data 1. [file elife-74519-data1.zip › Source data 1/Western blots/labelled/figure9C-labelled/figure9C-GAPDH-3-labelled.jpg]

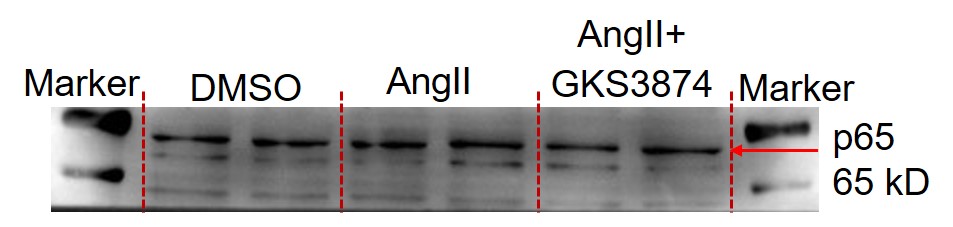

Supplement: Source data 1. [file elife-74519-data1.zip › Source data 1/Western blots/labelled/figure9C-labelled/figure9C-p65-1-labelled.jpg]

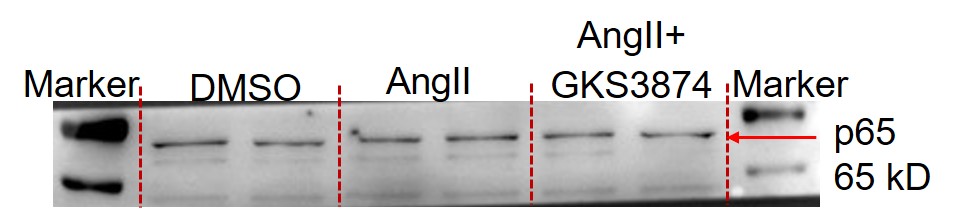

Supplement: Source data 1. [file elife-74519-data1.zip › Source data 1/Western blots/labelled/figure9C-labelled/figure9C-p65-2-labelled.jpg]

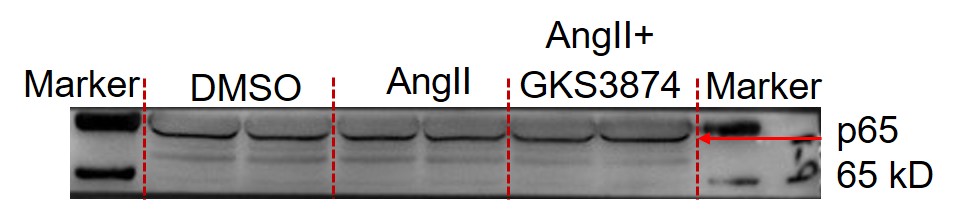

Supplement: Source data 1. [file elife-74519-data1.zip › Source data 1/Western blots/labelled/figure9C-labelled/figure9C-p65-3-labelled.jpg]

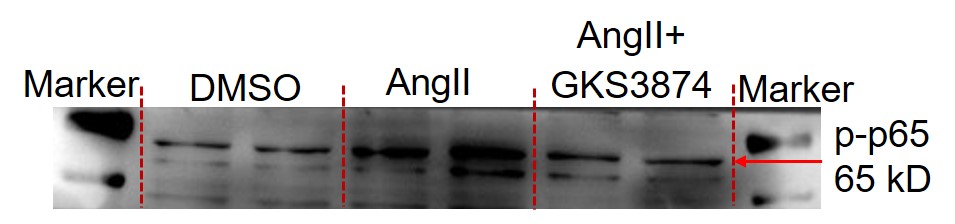

Supplement: Source data 1. [file elife-74519-data1.zip › Source data 1/Western blots/labelled/figure9C-labelled/figure9C-pp65-1-labelled.jpg]

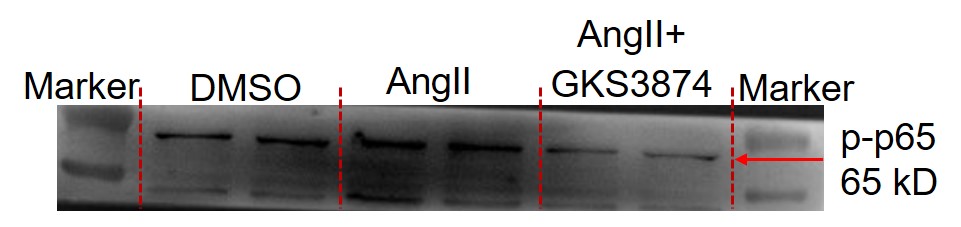

Supplement: Source data 1. [file elife-74519-data1.zip › Source data 1/Western blots/labelled/figure9C-labelled/figure9C-pp65-2-labelled.jpg]

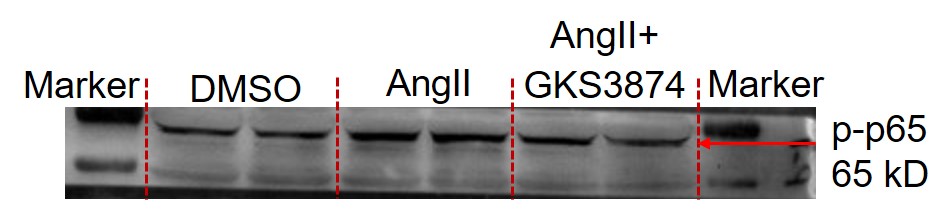

Supplement: Source data 1. [file elife-74519-data1.zip › Source data 1/Western blots/labelled/figure9C-labelled/figure9C-pp65-3-labelled.jpg]

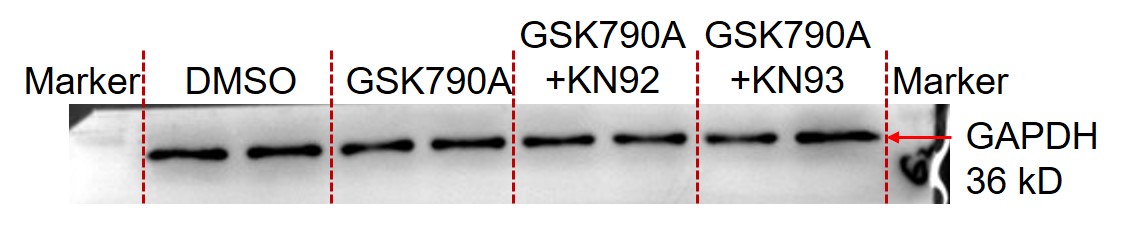

Supplement: Source data 1. [file elife-74519-data1.zip › Source data 1/Western blots/labelled/figure9E-labelled/figure9E-GAPDH-1-labelled.jpg]

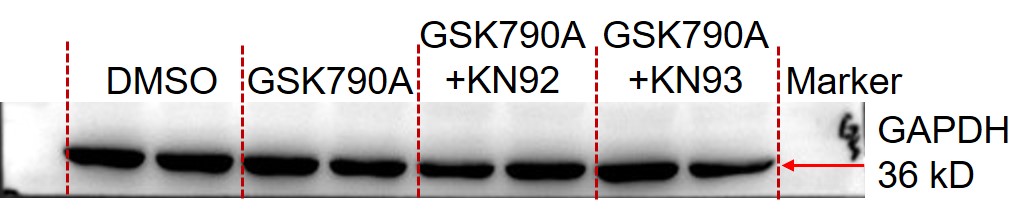

Supplement: Source data 1. [file elife-74519-data1.zip › Source data 1/Western blots/labelled/figure9E-labelled/figure9E-GAPDH-2-labelled.jpg]

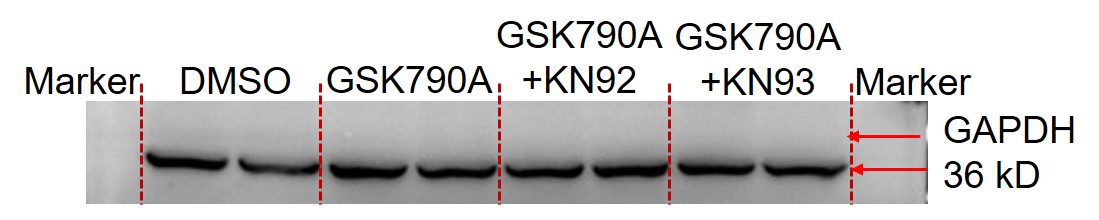

Supplement: Source data 1. [file elife-74519-data1.zip › Source data 1/Western blots/labelled/figure9E-labelled/figure9E-GAPDH-3-labelled.jpg]

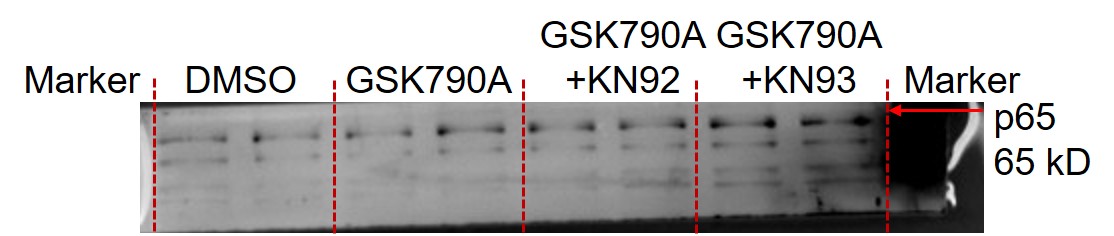

Supplement: Source data 1. [file elife-74519-data1.zip › Source data 1/Western blots/labelled/figure9E-labelled/figure9E-p65-1-labelled.jpg]

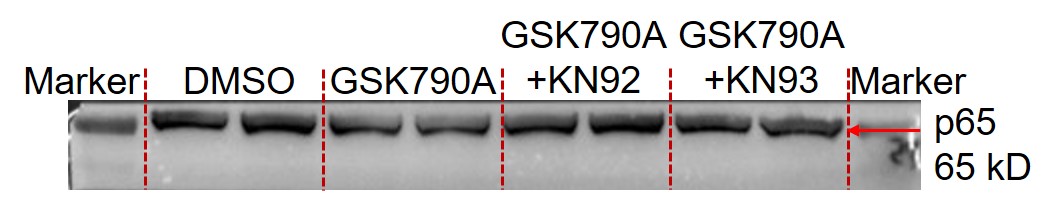

Supplement: Source data 1. [file elife-74519-data1.zip › Source data 1/Western blots/labelled/figure9E-labelled/figure9E-p65-3-labelled.jpg]

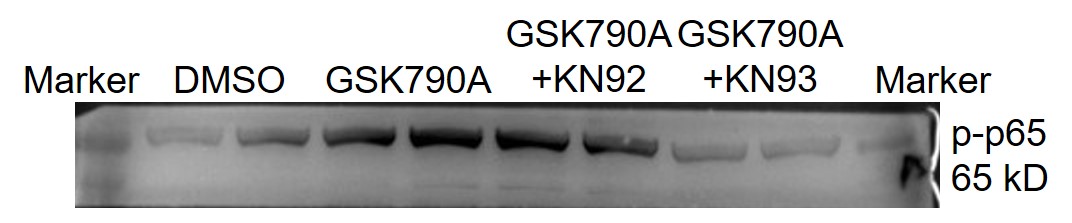

Supplement: Source data 1. [file elife-74519-data1.zip › Source data 1/Western blots/labelled/figure9E-labelled/figure9E-pp65-1-labelled.jpg]

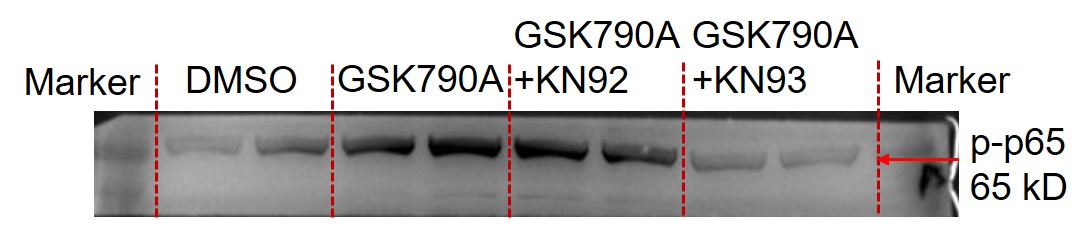

Supplement: Source data 1. [file elife-74519-data1.zip › Source data 1/Western blots/labelled/figure9E-labelled/figure9E-pp65-2-labelled.jpg]

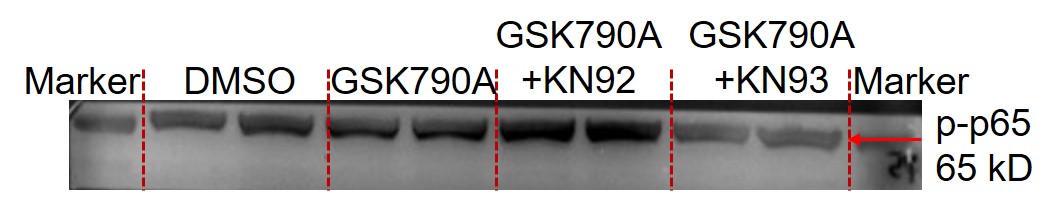

Supplement: Source data 1. [file elife-74519-data1.zip › Source data 1/Western blots/labelled/figure9E-labelled/figure9E-pp65-3-labelled.jpg]

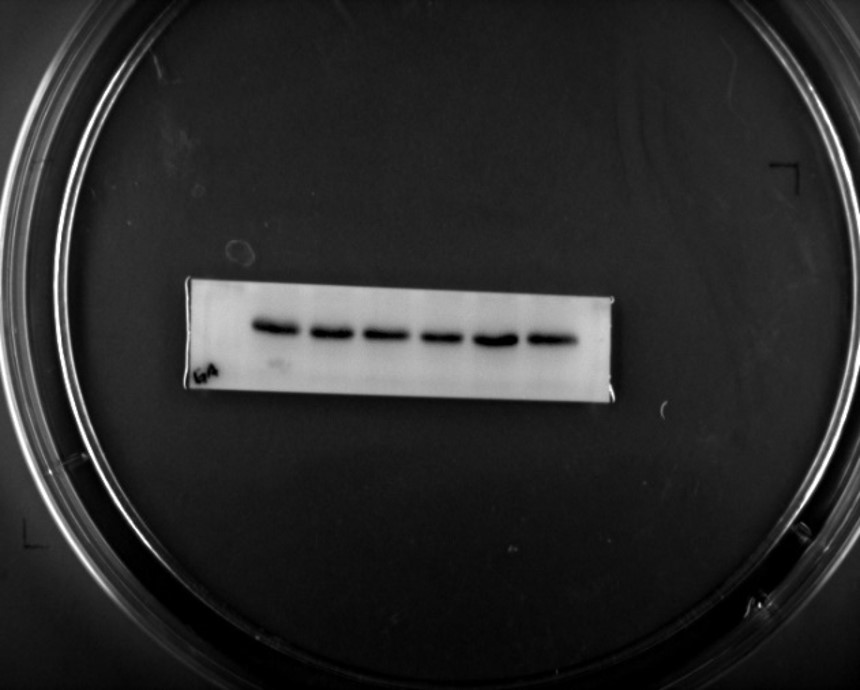

Supplement: Source data 1. [file elife-74519-data1.zip › Source data 1/Western blots/original/figure1A-original/WT-TAC1w-GAPDH-1-original.jpg]

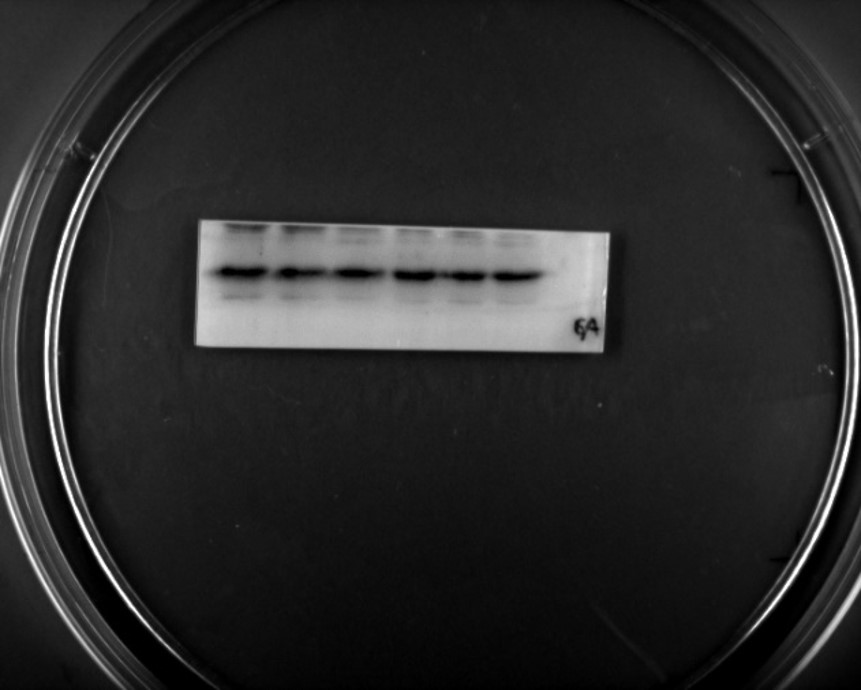

Supplement: Source data 1. [file elife-74519-data1.zip › Source data 1/Western blots/original/figure1A-original/WT-TAC1w-GAPDH-2-original.jpg]

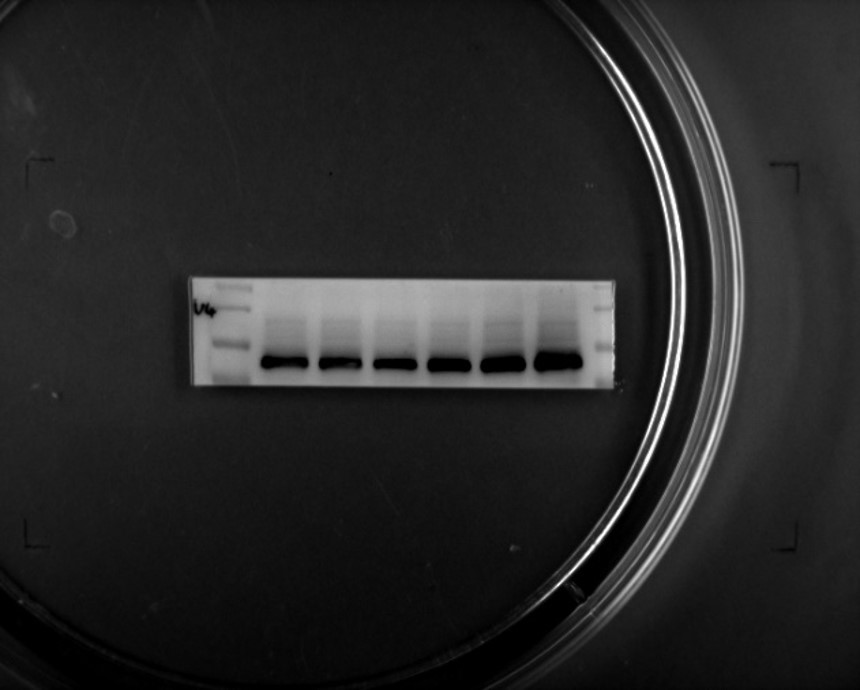

Supplement: Source data 1. [file elife-74519-data1.zip › Source data 1/Western blots/original/figure1A-original/WT-TAC1w-TRPV4-1-original.jpg]

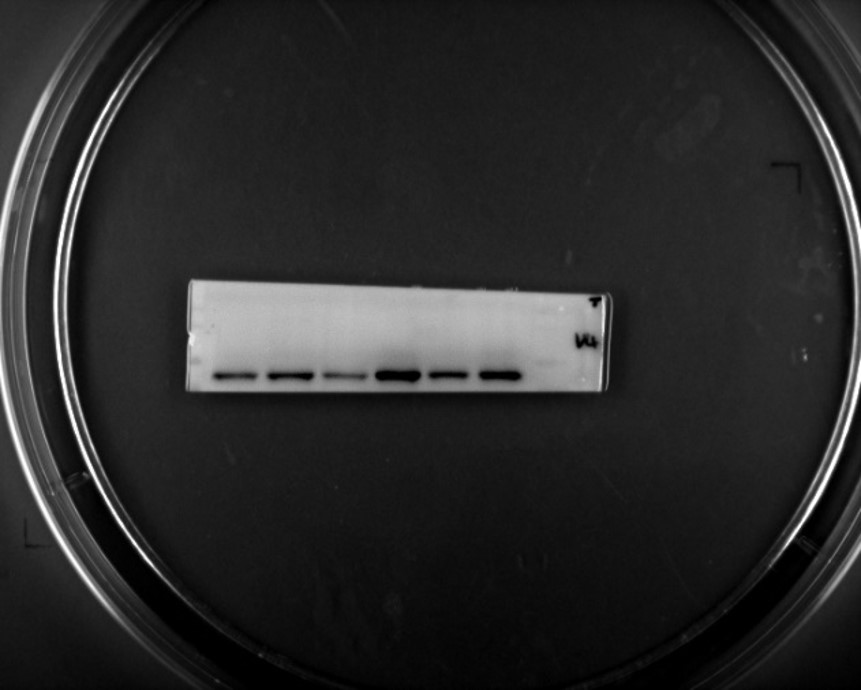

Supplement: Source data 1. [file elife-74519-data1.zip › Source data 1/Western blots/original/figure1A-original/WT-TAC1w-TRPV4-2-original.jpg]

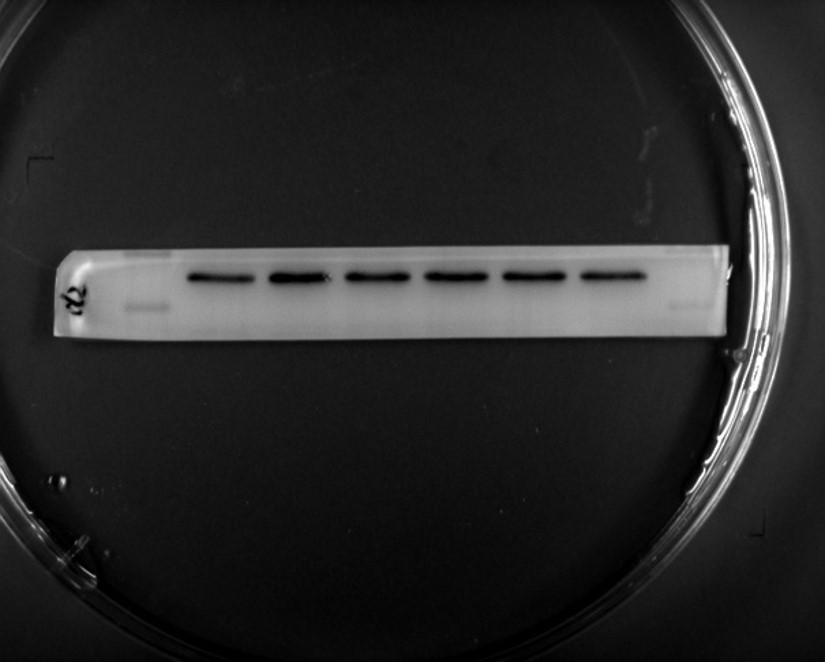

Supplement: Source data 1. [file elife-74519-data1.zip › Source data 1/Western blots/original/figure1A-original/WT-TAC2d-GAPDH-1-original.jpg]

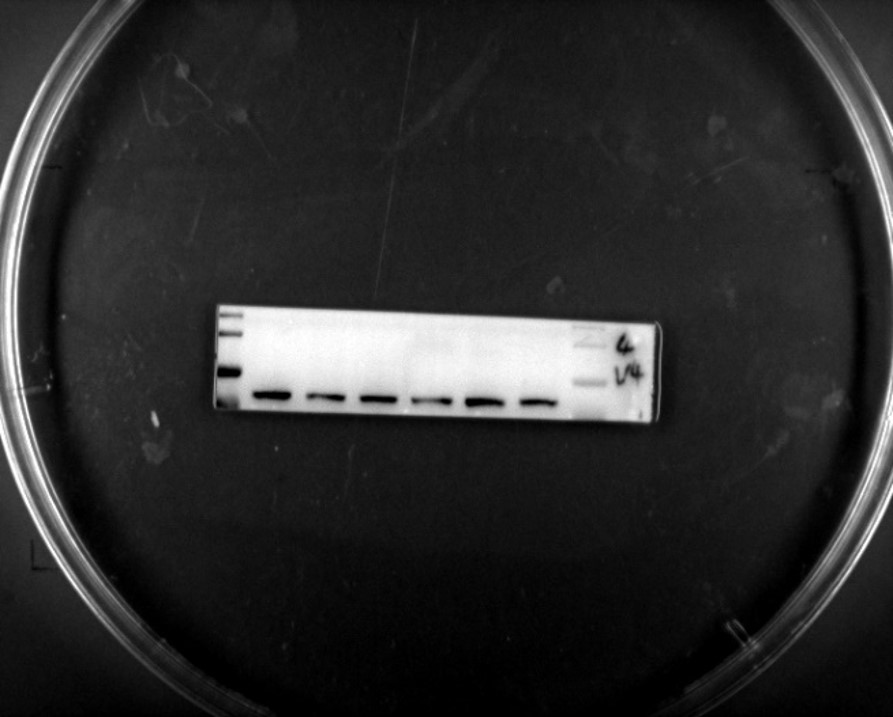

Supplement: Source data 1. [file elife-74519-data1.zip › Source data 1/Western blots/original/figure1A-original/WT-TAC2d-GAPDH-2-original.jpg]

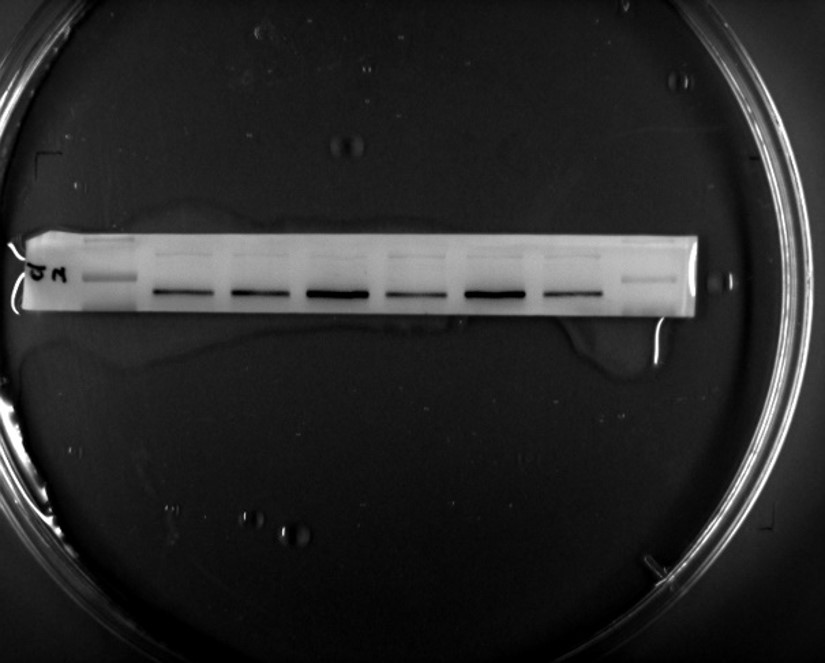

Supplement: Source data 1. [file elife-74519-data1.zip › Source data 1/Western blots/original/figure1A-original/WT-TAC2d-TRPV4-1-original.jpg]

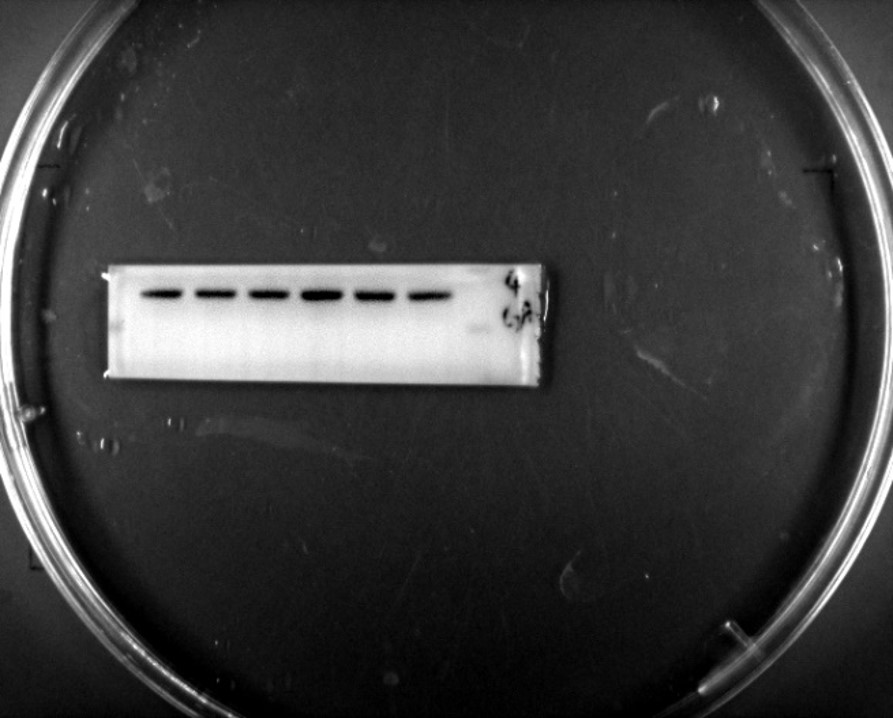

Supplement: Source data 1. [file elife-74519-data1.zip › Source data 1/Western blots/original/figure1A-original/WT-TAC2d-TRPV4-2-original.jpg]

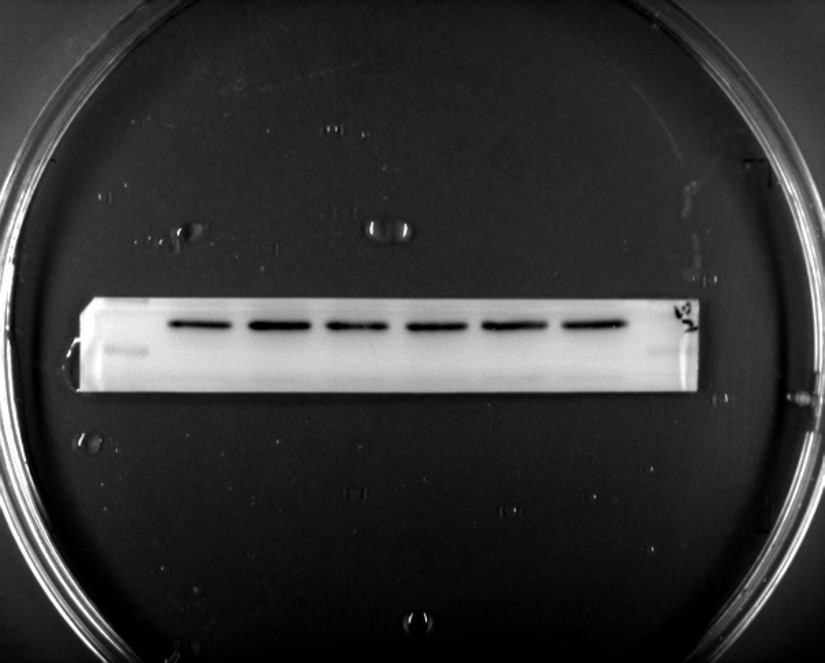

Supplement: Source data 1. [file elife-74519-data1.zip › Source data 1/Western blots/original/figure1A-original/WT-TAC2w-GAPDH-1-original.jpg]

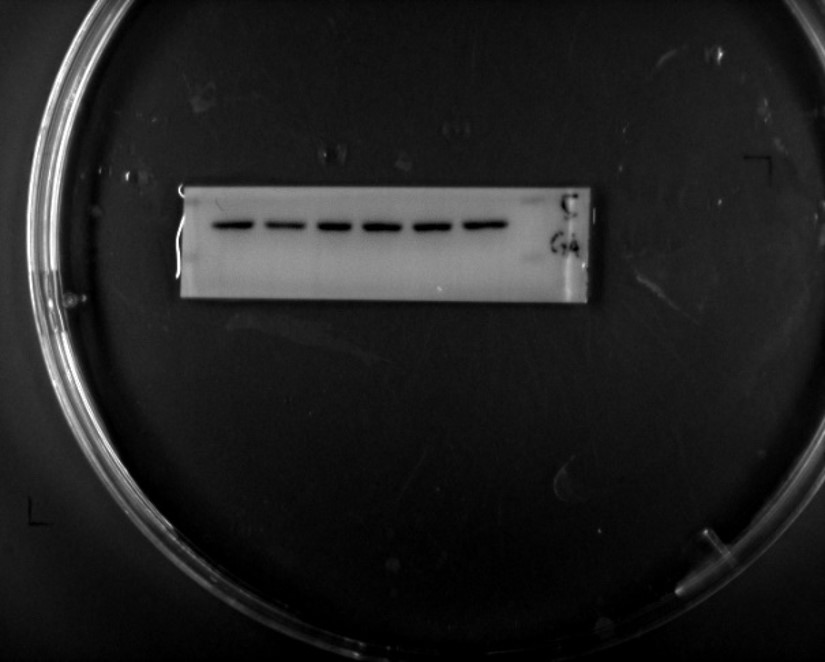

Supplement: Source data 1. [file elife-74519-data1.zip › Source data 1/Western blots/original/figure1A-original/WT-TAC2w-GAPDH-2-original.jpg]

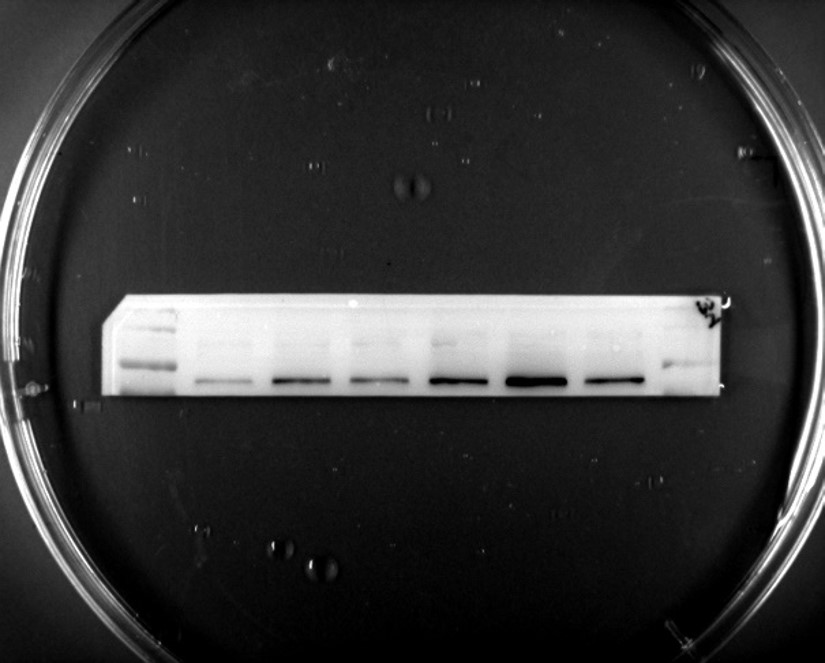

Supplement: Source data 1. [file elife-74519-data1.zip › Source data 1/Western blots/original/figure1A-original/WT-TAC2w-TRPV4-1-original.jpg]

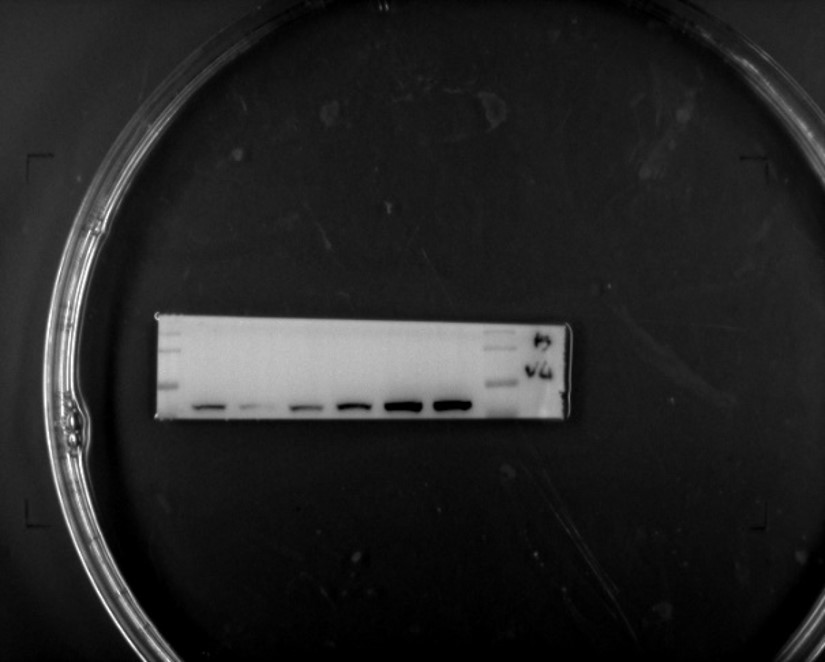

Supplement: Source data 1. [file elife-74519-data1.zip › Source data 1/Western blots/original/figure1A-original/WT-TAC2w-TRPV4-2-original.jpg]

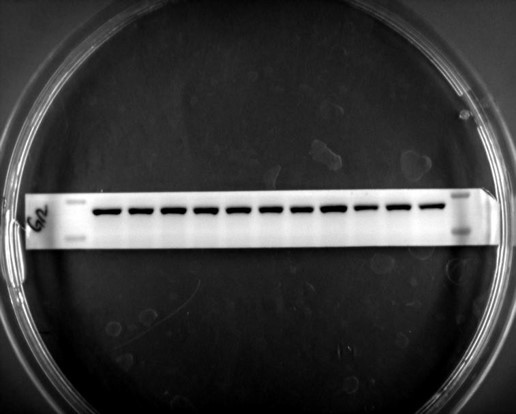

Supplement: Source data 1. [file elife-74519-data1.zip › Source data 1/Western blots/original/figure1A-original/WT-TAC4w-GAPDH-1-original.jpg]

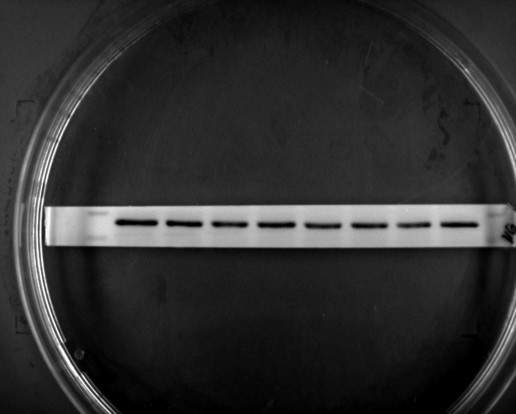

Supplement: Source data 1. [file elife-74519-data1.zip › Source data 1/Western blots/original/figure1A-original/WT-TAC4w-GAPDH-2-original.jpg]

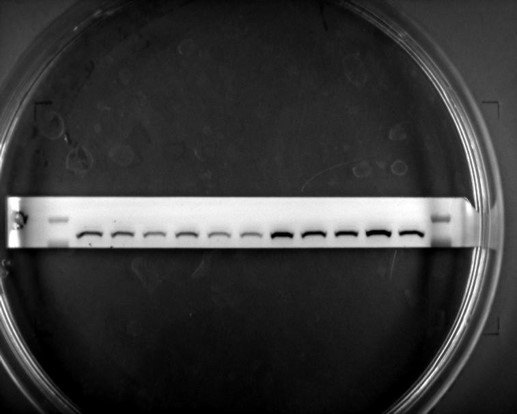

Supplement: Source data 1. [file elife-74519-data1.zip › Source data 1/Western blots/original/figure1A-original/WT-TAC4w-TRPV4-1-original.jpg]

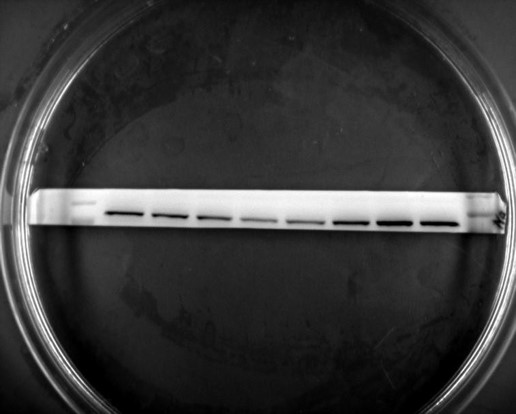

Supplement: Source data 1. [file elife-74519-data1.zip › Source data 1/Western blots/original/figure1A-original/WT-TAC4w-TRPV4-2-original.jpg]

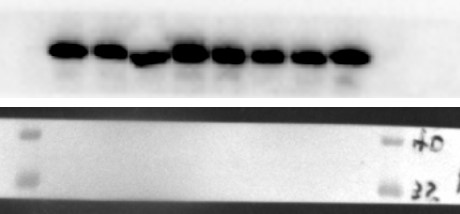

Supplement: Source data 1. [file elife-74519-data1.zip › Source data 1/Western blots/original/figure1D-original/human heart tissue- GAPDH-original.jpg]

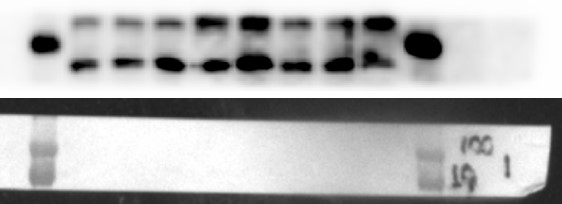

Supplement: Source data 1. [file elife-74519-data1.zip › Source data 1/Western blots/original/figure1D-original/human heart tissue- TRPV4-original.jpg]

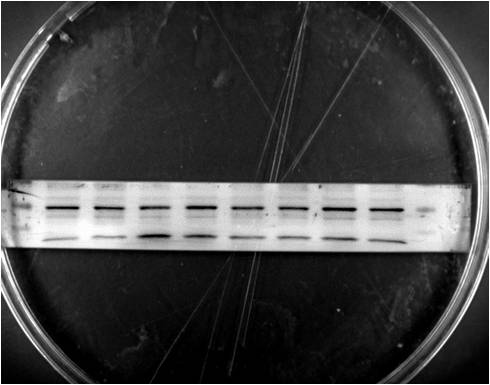

Supplement: Source data 1. [file elife-74519-data1.zip › Source data 1/Western blots/original/figure4A-original/figure4A-IL-1β-1-original.jpg]

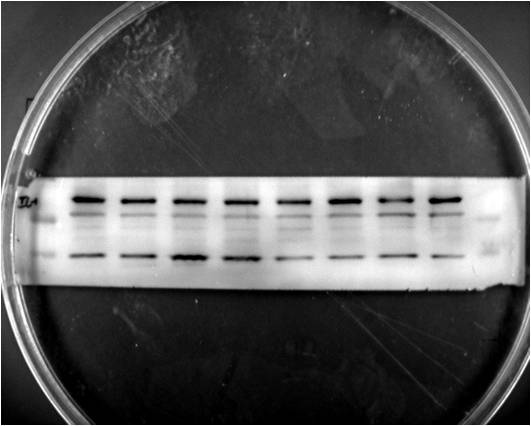

Supplement: Source data 1. [file elife-74519-data1.zip › Source data 1/Western blots/original/figure4A-original/figure4A-IL-1β-2-original.jpg]

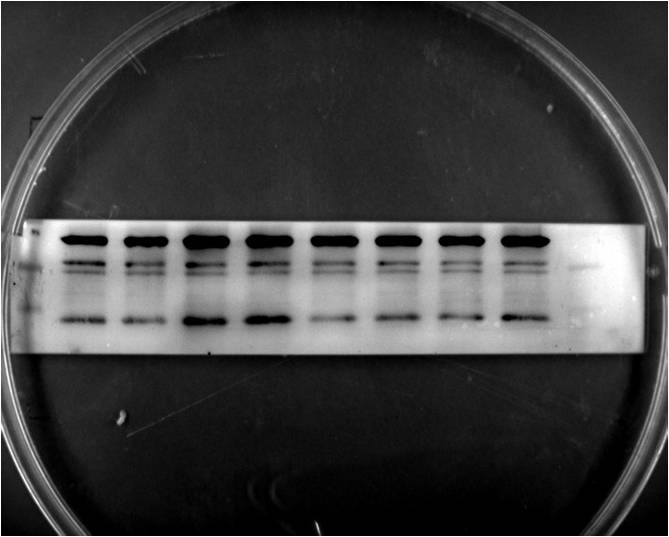

Supplement: Source data 1. [file elife-74519-data1.zip › Source data 1/Western blots/original/figure4A-original/figure4A-IL-1β-3-original.jpg]

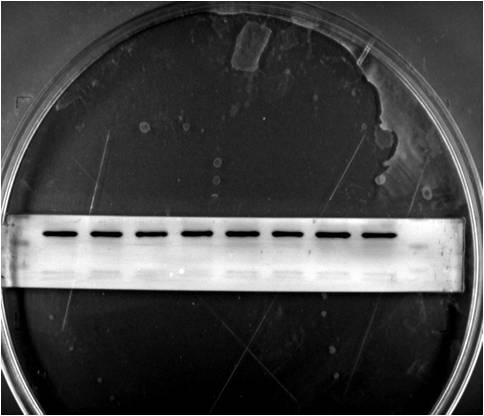

Supplement: Source data 1. [file elife-74519-data1.zip › Source data 1/Western blots/original/figure4A-original/figure4A-IL1β-GAPDH-1-original.jpg]

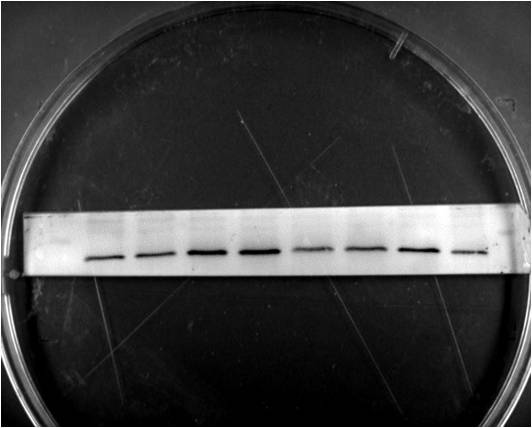

Supplement: Source data 1. [file elife-74519-data1.zip › Source data 1/Western blots/original/figure4A-original/figure4A-IL6-1-original.jpg]

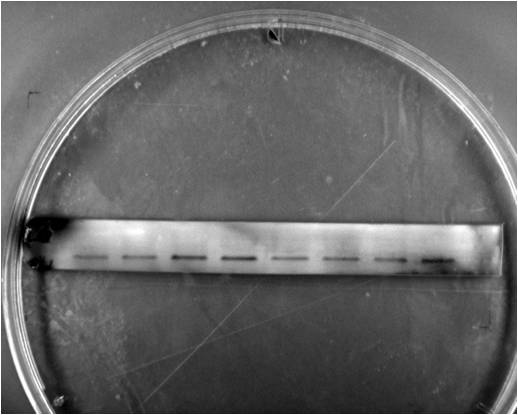

Supplement: Source data 1. [file elife-74519-data1.zip › Source data 1/Western blots/original/figure4A-original/figure4A-IL6-2-original.jpg]

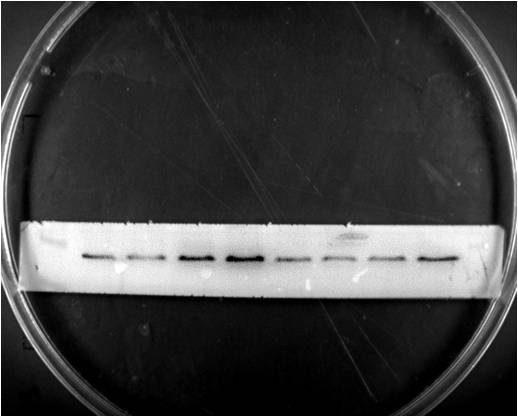

Supplement: Source data 1. [file elife-74519-data1.zip › Source data 1/Western blots/original/figure4A-original/figure4A-IL6-3-original.jpg]

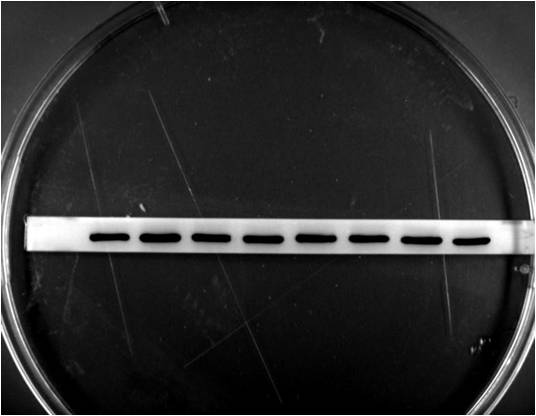

Supplement: Source data 1. [file elife-74519-data1.zip › Source data 1/Western blots/original/figure4A-original/figure4A-IL6-GAPDH-1-original.jpg]

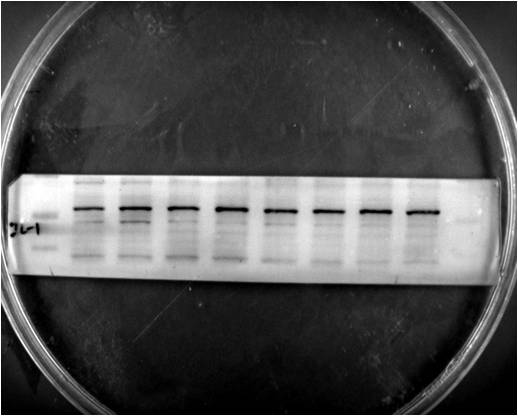

Supplement: Source data 1. [file elife-74519-data1.zip › Source data 1/Western blots/original/figure4A-original/figure4A-IL6-GAPDH-2-original.jpg]

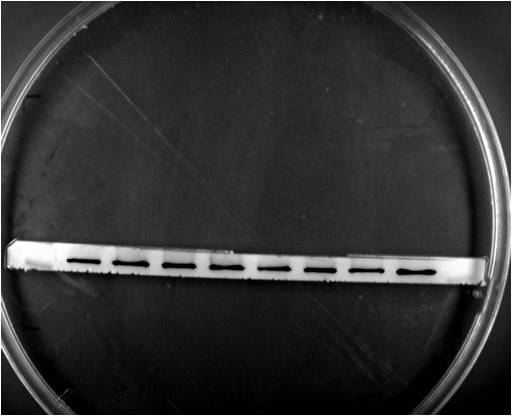

Supplement: Source data 1. [file elife-74519-data1.zip › Source data 1/Western blots/original/figure4A-original/figure4A-IL6-GAPDH-3-original.jpg]

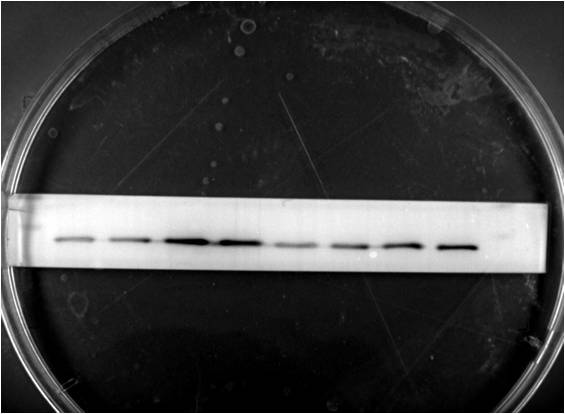

Supplement: Source data 1. [file elife-74519-data1.zip › Source data 1/Western blots/original/figure4A-original/figure4A-TNFα-1-original.jpg]

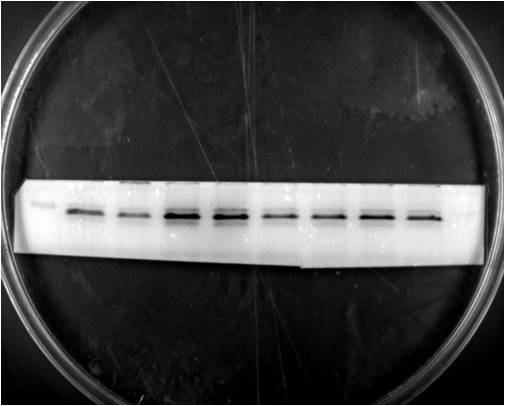

Supplement: Source data 1. [file elife-74519-data1.zip › Source data 1/Western blots/original/figure4A-original/figure4A-TNFα-2-original.jpg]

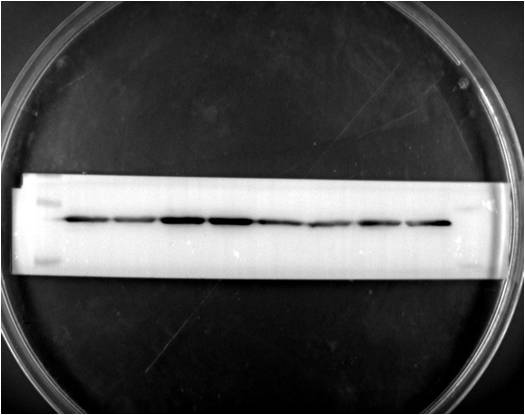

Supplement: Source data 1. [file elife-74519-data1.zip › Source data 1/Western blots/original/figure4A-original/figure4A-TNFα-3-original.jpg]

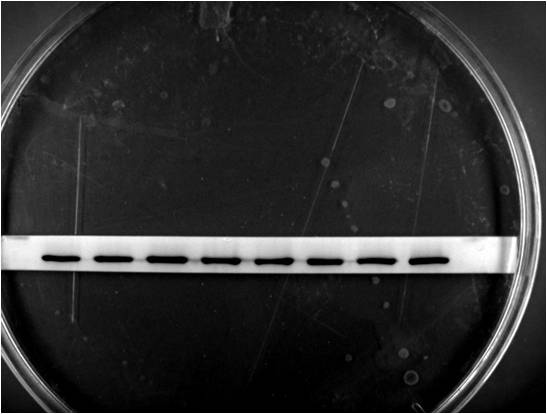

Supplement: Source data 1. [file elife-74519-data1.zip › Source data 1/Western blots/original/figure4A-original/figure4A-TNFα-GAPDH-1-origina.jpg]

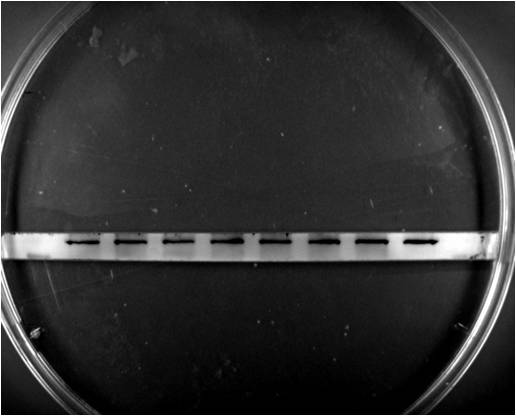

Supplement: Source data 1. [file elife-74519-data1.zip › Source data 1/Western blots/original/figure4A-original/figure4A-TNFα-GAPDH-2-origina.jpg]

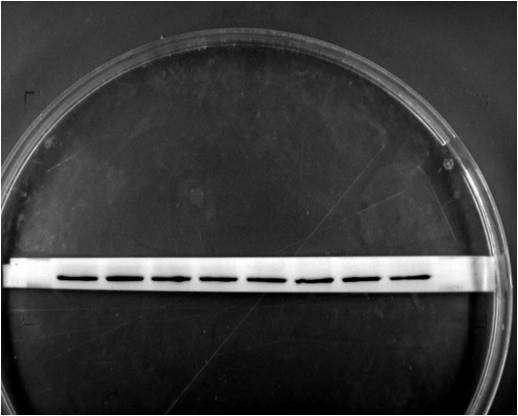

Supplement: Source data 1. [file elife-74519-data1.zip › Source data 1/Western blots/original/figure4A-original/figure4A-TNFα-GAPDH-3-original.jpg]

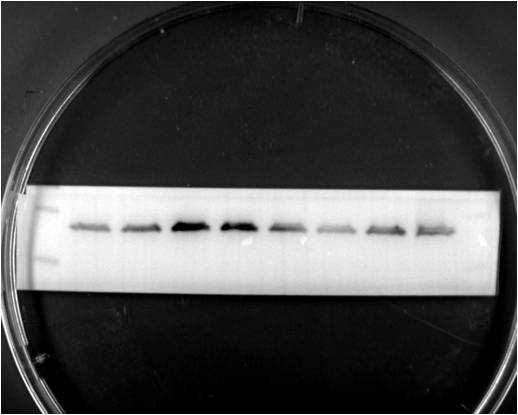

Supplement: Source data 1. [file elife-74519-data1.zip › Source data 1/Western blots/original/figure4J-original/figure4J-ASC-2-original.jpg]

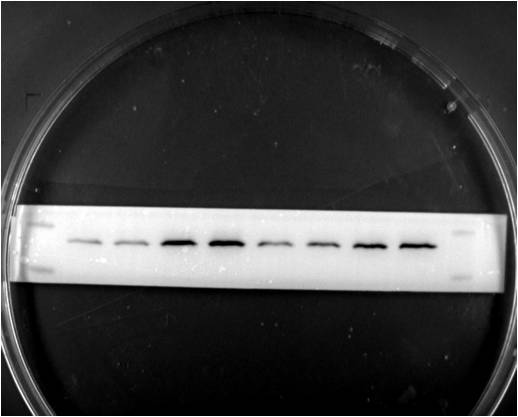

Supplement: Source data 1. [file elife-74519-data1.zip › Source data 1/Western blots/original/figure4J-original/figure4J-ASC-3-original.jpg]

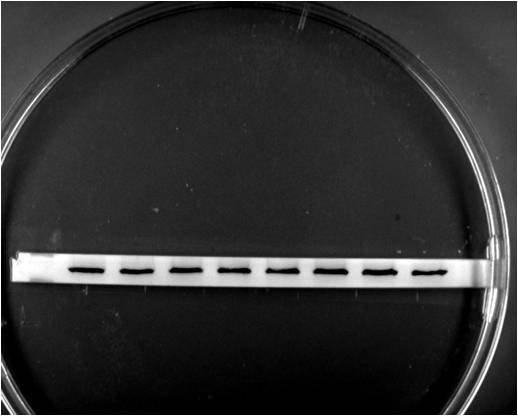

Supplement: Source data 1. [file elife-74519-data1.zip › Source data 1/Western blots/original/figure4J-original/figure4J-ASC-GAPDH-2-original.jpg]

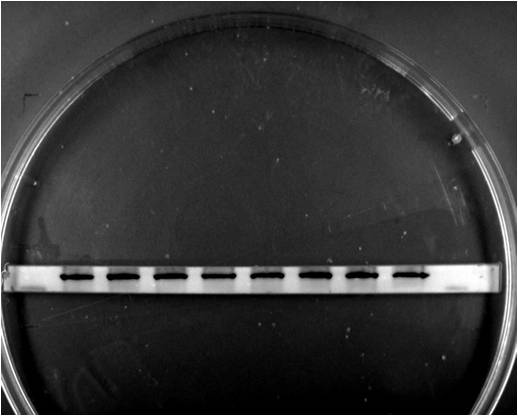

Supplement: Source data 1. [file elife-74519-data1.zip › Source data 1/Western blots/original/figure4J-original/figure4J-ASC-GAPDH-3-original.jpg]

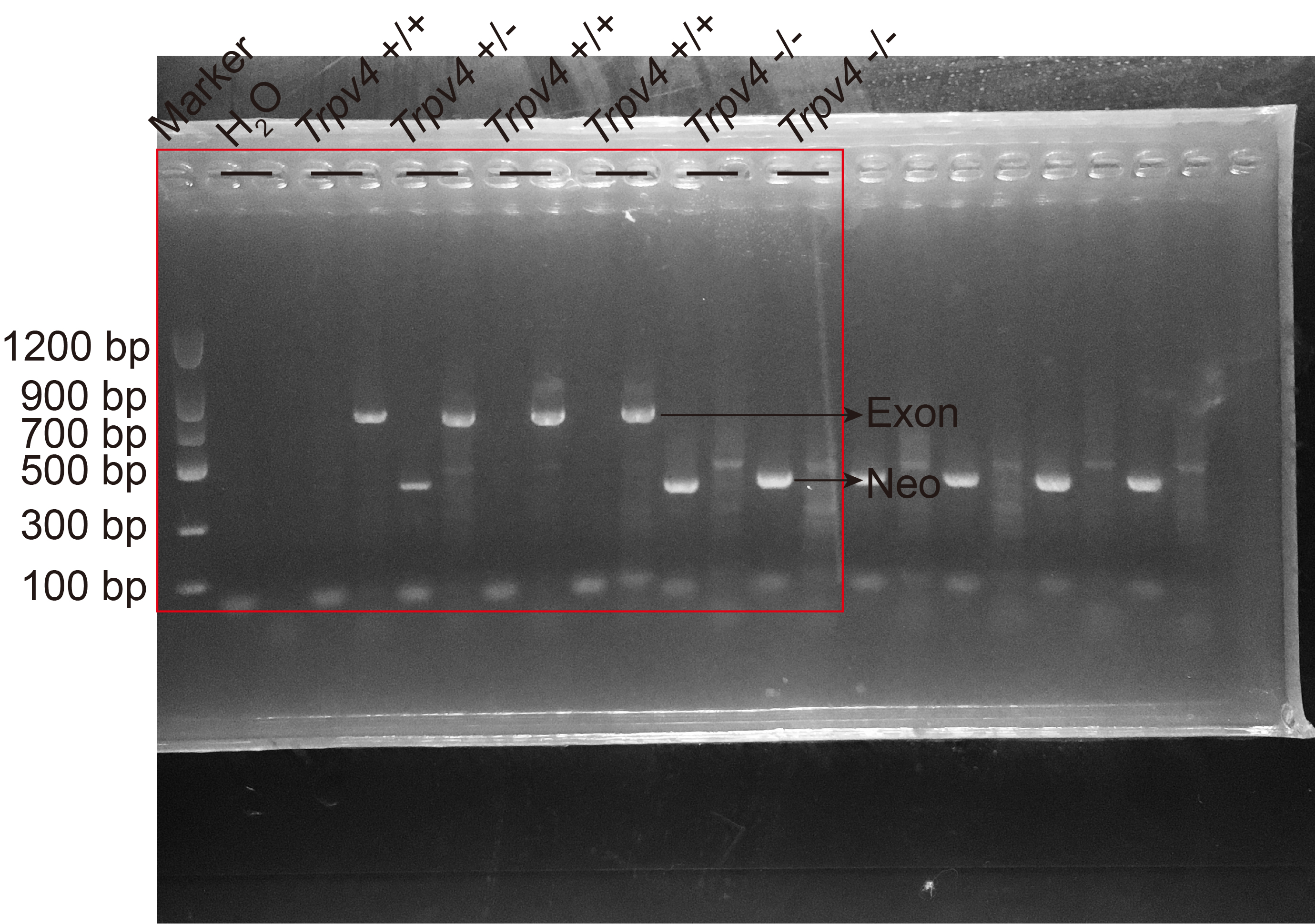

Supplement: Appendix 1—figure 1—source data 1. [file elife-74519-app1-fig1-data1.zip › appendix1-figure 1 source data/gel-labelled/appendix1-figure1A-labelled.jpg]

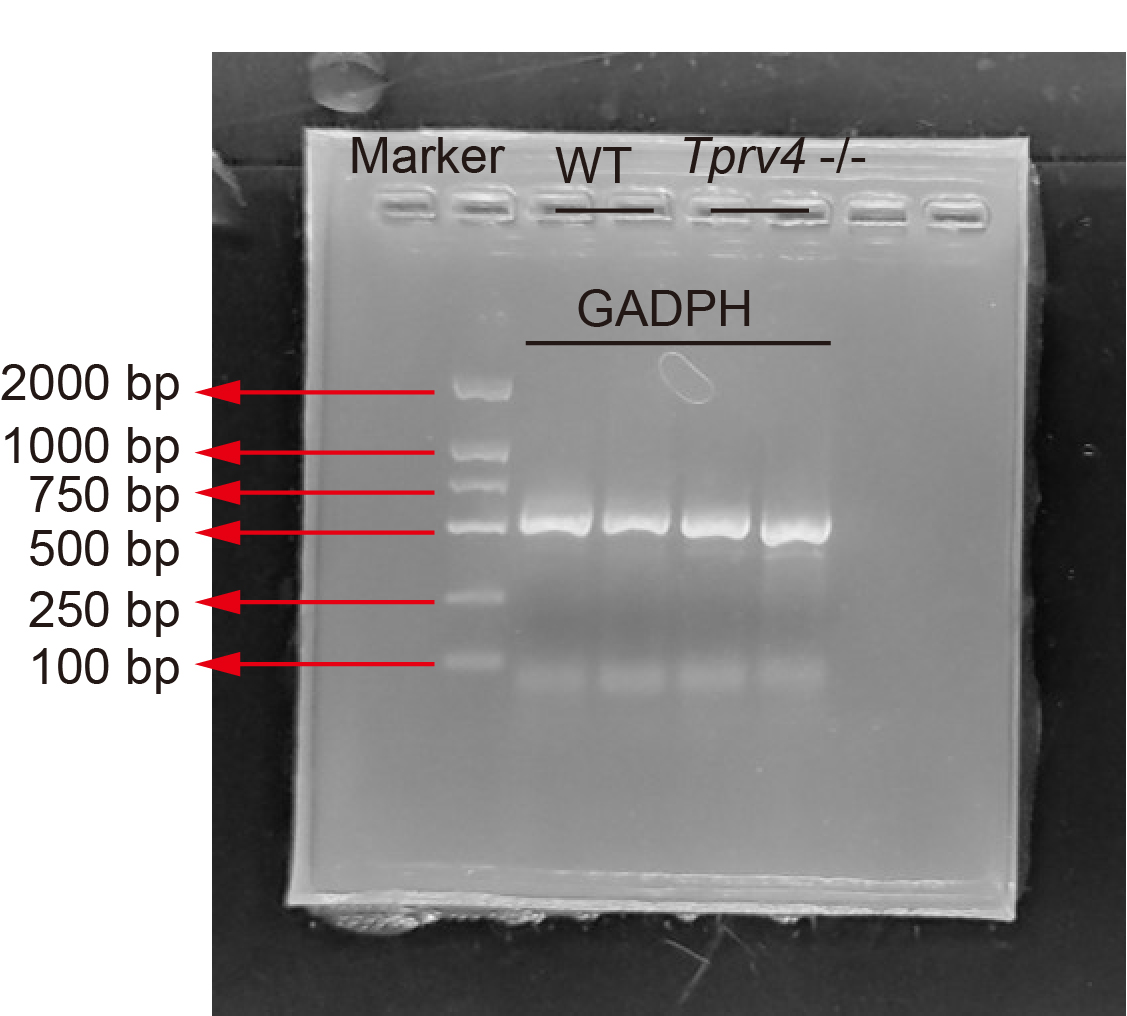

Supplement: Appendix 1—figure 1—source data 1. [file elife-74519-app1-fig1-data1.zip › appendix1-figure 1 source data/gel-labelled/appendix1-figure1B-GAPDH-labelled.jpg]

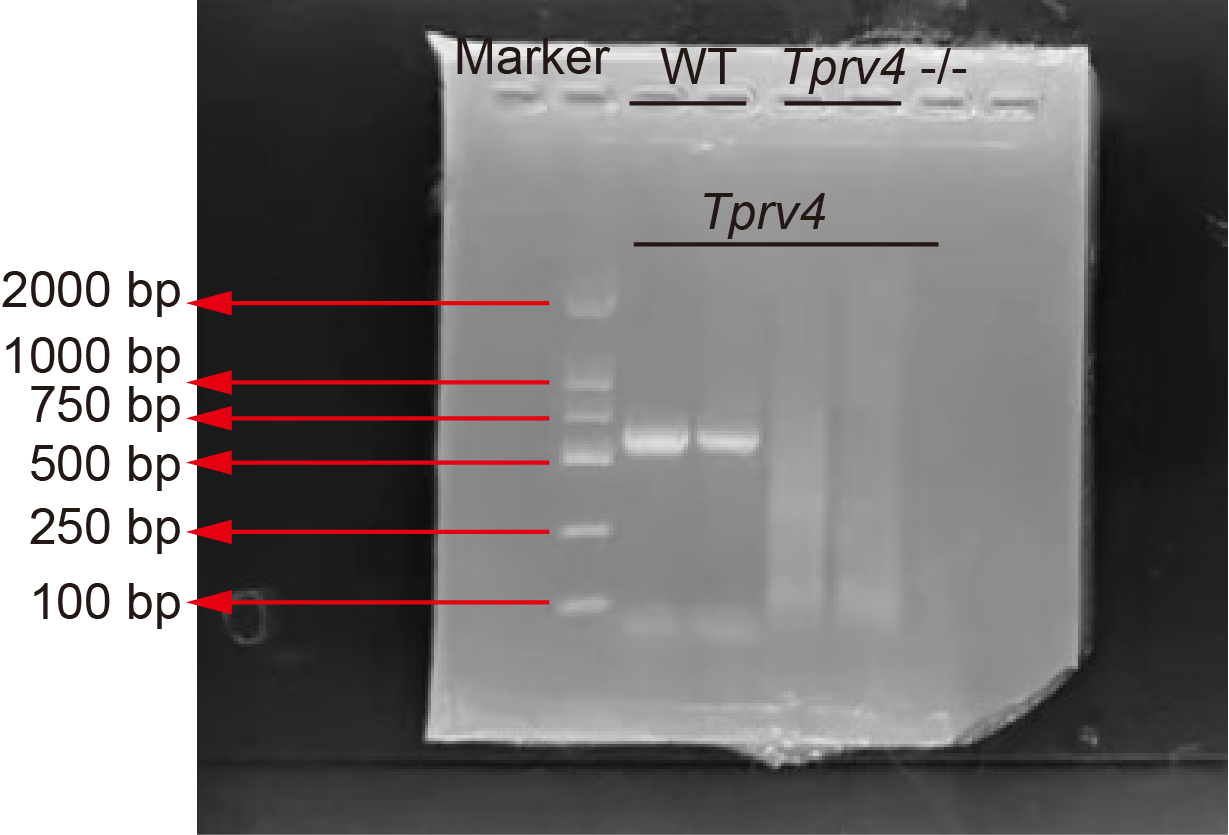

Supplement: Appendix 1—figure 1—source data 1. [file elife-74519-app1-fig1-data1.zip › appendix1-figure 1 source data/gel-labelled/appendix1-figure1B-Trpv4-labelled.jpg]

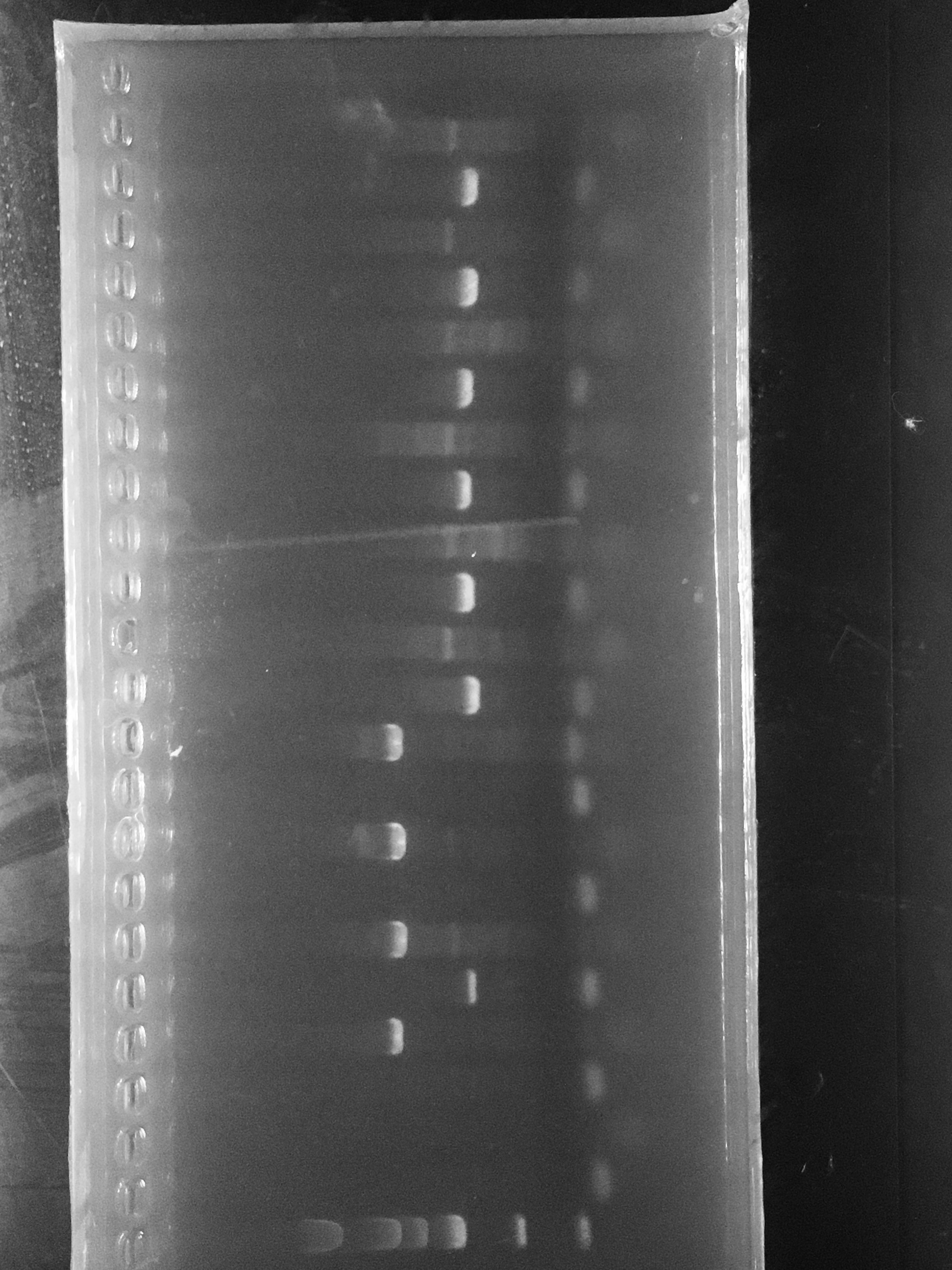

Supplement: Appendix 1—figure 1—source data 1. [file elife-74519-app1-fig1-data1.zip › appendix1-figure 1 source data/gel-original/appendix1-figure1A-original.jpg]

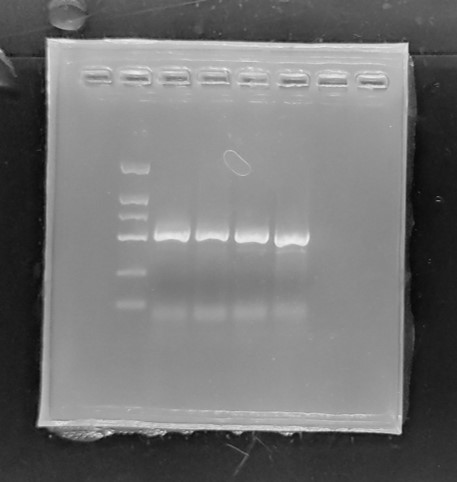

Supplement: Appendix 1—figure 1—source data 1. [file elife-74519-app1-fig1-data1.zip › appendix1-figure 1 source data/gel-original/appendix1-figure1B-GAPDH-original.jpg]

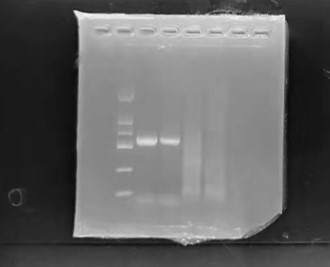

Supplement: Appendix 1—figure 1—source data 1. [file elife-74519-app1-fig1-data1.zip › appendix1-figure 1 source data/gel-original/appendix1-figure1B-Trpv4-original.jpg]
