# Supplementary figures and images for: Activation of transient receptor potential vanilloid 4 is involved in pressure overload-induced cardiac hypertrophy (part 1 of 2)
Source: eLife. 2022 Jun 22;11:e74519. doi: 10.7554/eLife.74519 (PMC9224988; doi:10.7554/eLife.74519)

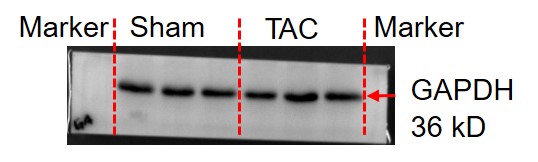

Supplement: Source data 1. [file elife-74519-data1.zip › Source data 1/Western blots/labelled/figure1A-labelled/WT-TAC1w-GAPDH1-labelled.jpg]

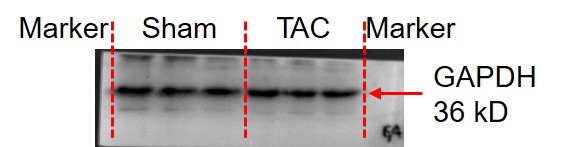

Supplement: Source data 1. [file elife-74519-data1.zip › Source data 1/Western blots/labelled/figure1A-labelled/WT-TAC1w-GAPDH2-labelled.jpg]

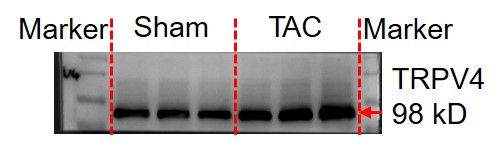

Supplement: Source data 1. [file elife-74519-data1.zip › Source data 1/Western blots/labelled/figure1A-labelled/WT-TAC1w-TRPV4-1-labelled.jpg]

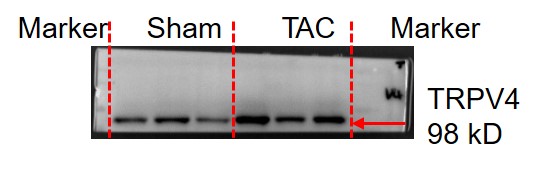

Supplement: Source data 1. [file elife-74519-data1.zip › Source data 1/Western blots/labelled/figure1A-labelled/WT-TAC1w-TRPV4-2-labelled.jpg]

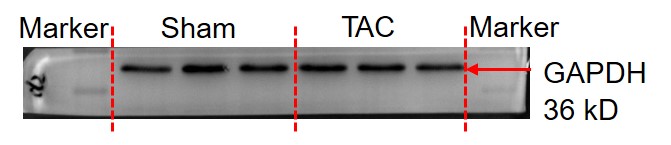

Supplement: Source data 1. [file elife-74519-data1.zip › Source data 1/Western blots/labelled/figure1A-labelled/WT-TAC2d-GAPDH 1-labelled.jpg]

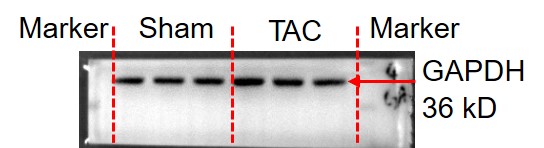

Supplement: Source data 1. [file elife-74519-data1.zip › Source data 1/Western blots/labelled/figure1A-labelled/WT-TAC2d-GAPDH 2-labelled.jpg]

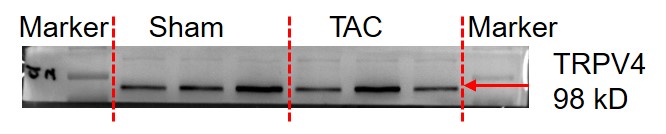

Supplement: Source data 1. [file elife-74519-data1.zip › Source data 1/Western blots/labelled/figure1A-labelled/WT-TAC2d-TRPV4 1-labelled.jpg]

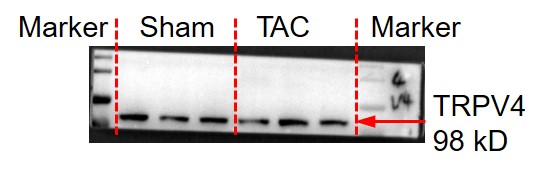

Supplement: Source data 1. [file elife-74519-data1.zip › Source data 1/Western blots/labelled/figure1A-labelled/WT-TAC2d-TRPV4 2-labelled.jpg]

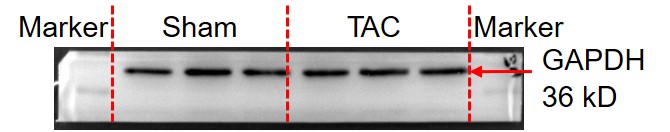

Supplement: Source data 1. [file elife-74519-data1.zip › Source data 1/Western blots/labelled/figure1A-labelled/WT-TAC2w-GAPDH-1 labelled.jpg]

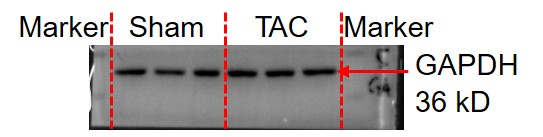

Supplement: Source data 1. [file elife-74519-data1.zip › Source data 1/Western blots/labelled/figure1A-labelled/WT-TAC2w-GAPDH-2 labelled.jpg]

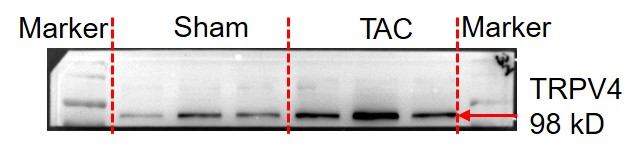

Supplement: Source data 1. [file elife-74519-data1.zip › Source data 1/Western blots/labelled/figure1A-labelled/WT-TAC2w-TRPV4-1 labelled.jpg]

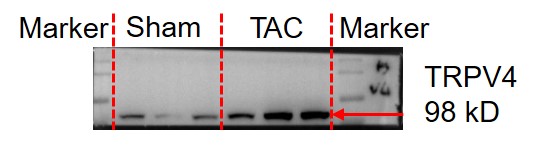

Supplement: Source data 1. [file elife-74519-data1.zip › Source data 1/Western blots/labelled/figure1A-labelled/WT-TAC2w-TRPV4-2 labelled.jpg]

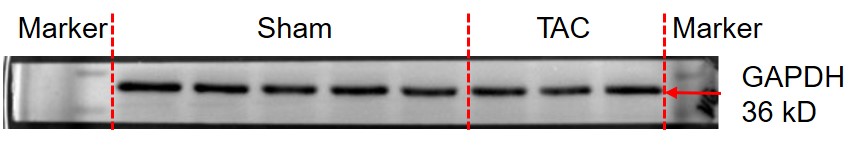

Supplement: Source data 1. [file elife-74519-data1.zip › Source data 1/Western blots/labelled/figure1A-labelled/WT-TAC4w-GAPDH-1-labelled.jpg]

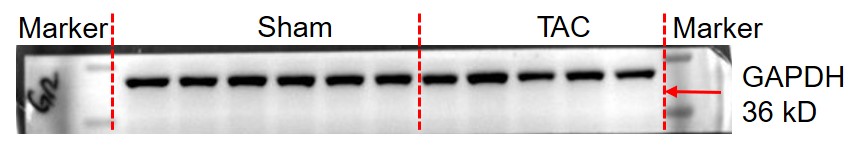

Supplement: Source data 1. [file elife-74519-data1.zip › Source data 1/Western blots/labelled/figure1A-labelled/WT-TAC4w-GAPDH-2-labelled.jpg]

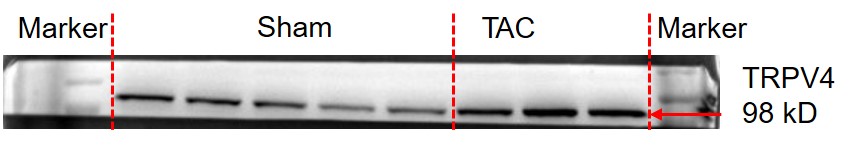

Supplement: Source data 1. [file elife-74519-data1.zip › Source data 1/Western blots/labelled/figure1A-labelled/WT-TAC4w-TRPV4-1-labelled.jpg]

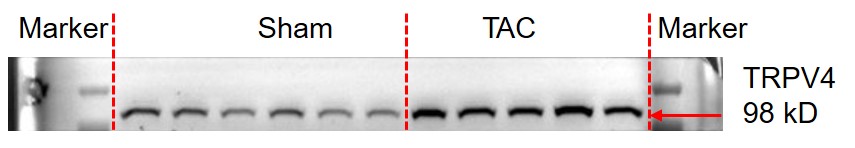

Supplement: Source data 1. [file elife-74519-data1.zip › Source data 1/Western blots/labelled/figure1A-labelled/WT-TAC4w-TRPV4-2-labelled.jpg]

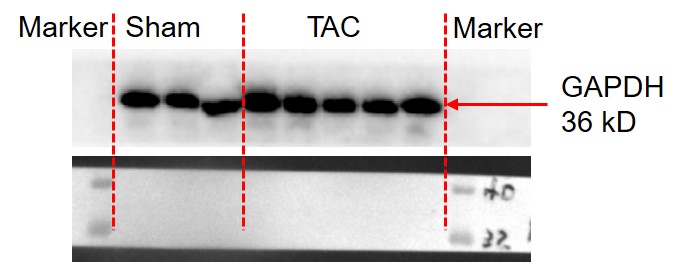

Supplement: Source data 1. [file elife-74519-data1.zip › Source data 1/Western blots/labelled/figure1D-labelled/human heart tissue-GAPDH-labbelled.jpg]

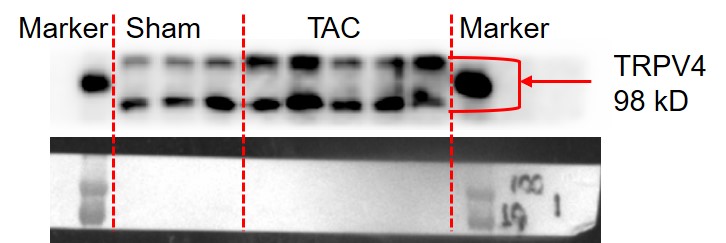

Supplement: Source data 1. [file elife-74519-data1.zip › Source data 1/Western blots/labelled/figure1D-labelled/human heart tissue-TRPV4-labbelled.jpg]

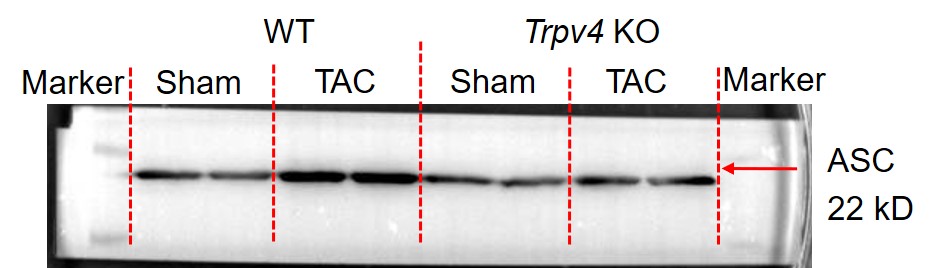

Supplement: Source data 1. [file elife-74519-data1.zip › Source data 1/Western blots/labelled/figure4A-labelled/figure4A-ASC-1-labelled.jpg]

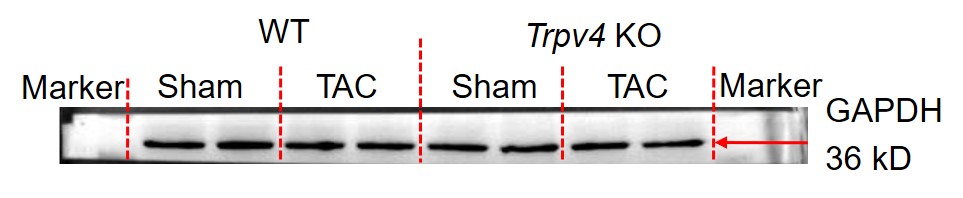

Supplement: Source data 1. [file elife-74519-data1.zip › Source data 1/Western blots/labelled/figure4A-labelled/figure4A-ASC-GAPDH-1-labelled.jpg]

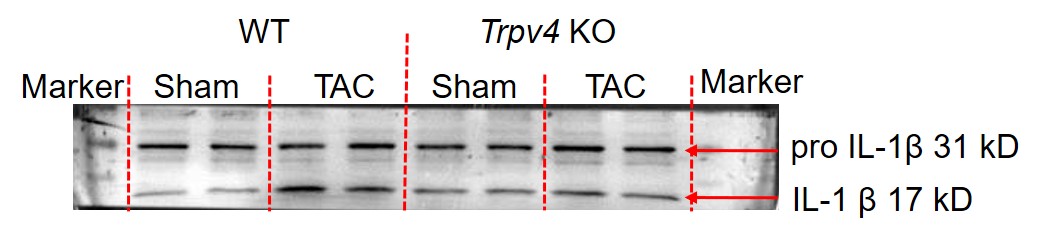

Supplement: Source data 1. [file elife-74519-data1.zip › Source data 1/Western blots/labelled/figure4A-labelled/figure4A-IL 1β-1-labelled.jpg]

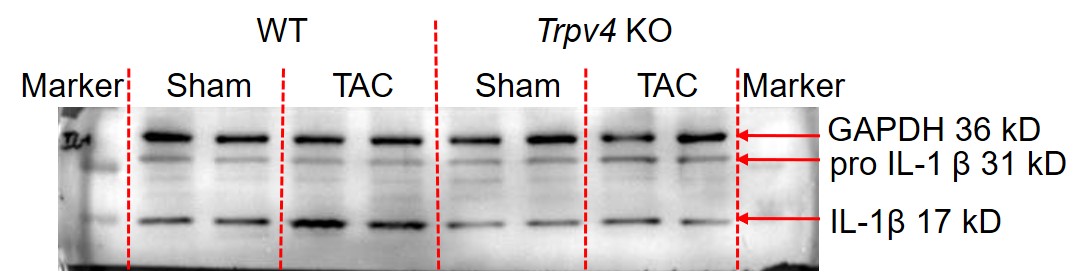

Supplement: Source data 1. [file elife-74519-data1.zip › Source data 1/Western blots/labelled/figure4A-labelled/figure4A-IL 1β-2-labelled.jpg]

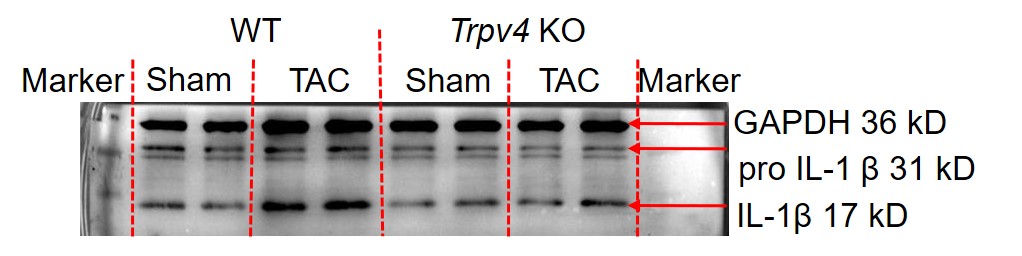

Supplement: Source data 1. [file elife-74519-data1.zip › Source data 1/Western blots/labelled/figure4A-labelled/figure4A-IL 1β-3-labelled.jpg]

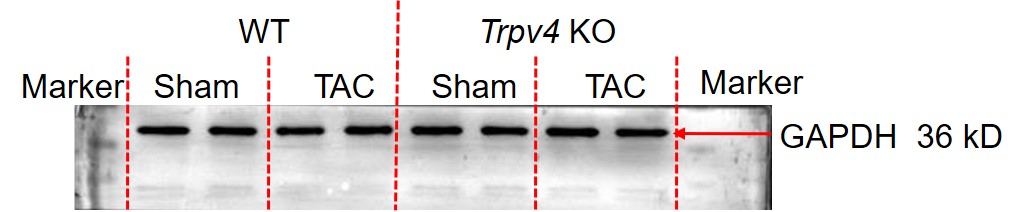

Supplement: Source data 1. [file elife-74519-data1.zip › Source data 1/Western blots/labelled/figure4A-labelled/figure4A-IL 1β-GAPDH-1-labelled.jpg]

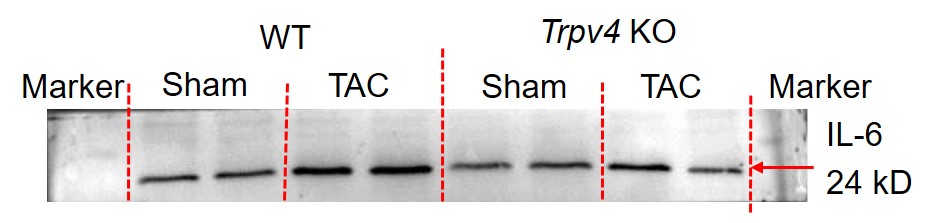

Supplement: Source data 1. [file elife-74519-data1.zip › Source data 1/Western blots/labelled/figure4A-labelled/figure4A-IL6-1-labelled.jpg]

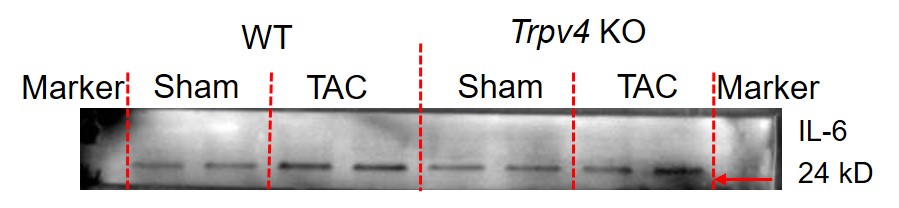

Supplement: Source data 1. [file elife-74519-data1.zip › Source data 1/Western blots/labelled/figure4A-labelled/figure4A-IL6-2-labelled.jpg]

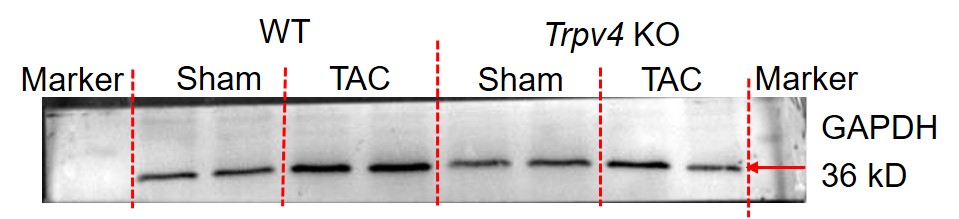

Supplement: Source data 1. [file elife-74519-data1.zip › Source data 1/Western blots/labelled/figure4A-labelled/figure4A-IL6-GAPDH-1-labelled.jpg]

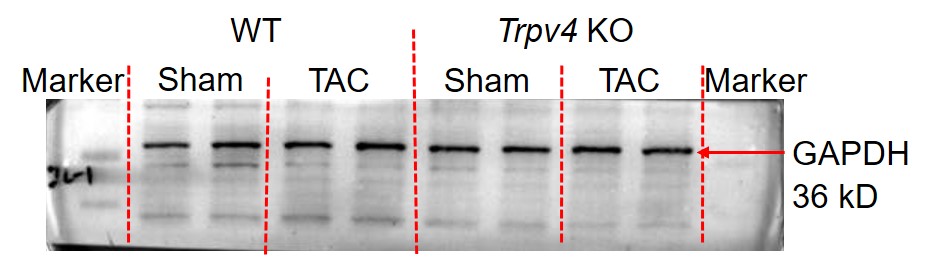

Supplement: Source data 1. [file elife-74519-data1.zip › Source data 1/Western blots/labelled/figure4A-labelled/figure4A-IL6-GAPDH-2-labelled.jpg]

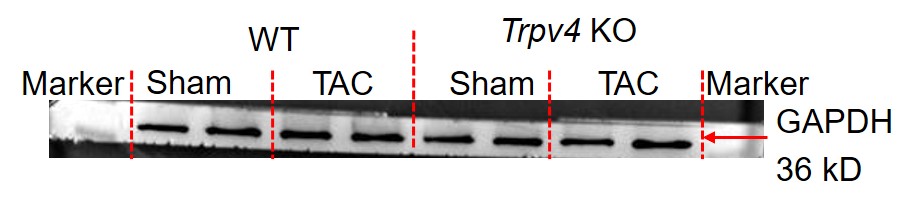

Supplement: Source data 1. [file elife-74519-data1.zip › Source data 1/Western blots/labelled/figure4A-labelled/figure4A-IL6-GAPDH-3-labelled.jpg]

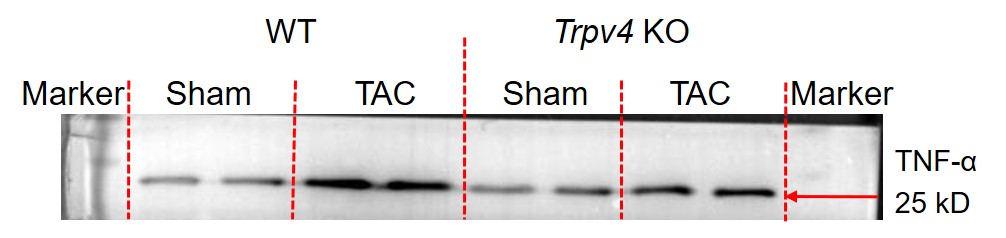

Supplement: Source data 1. [file elife-74519-data1.zip › Source data 1/Western blots/labelled/figure4A-labelled/figure4A-TNF α-1-labelled.jpg]

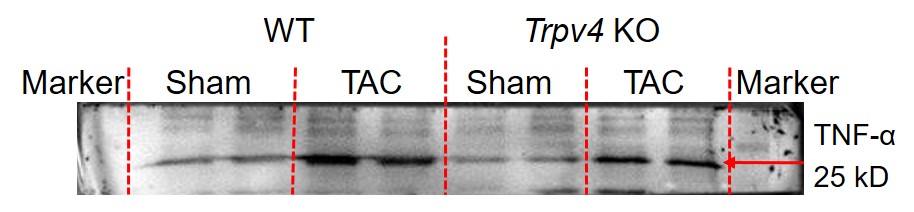

Supplement: Source data 1. [file elife-74519-data1.zip › Source data 1/Western blots/labelled/figure4A-labelled/figure4A-TNF α-2-labelled.jpg]

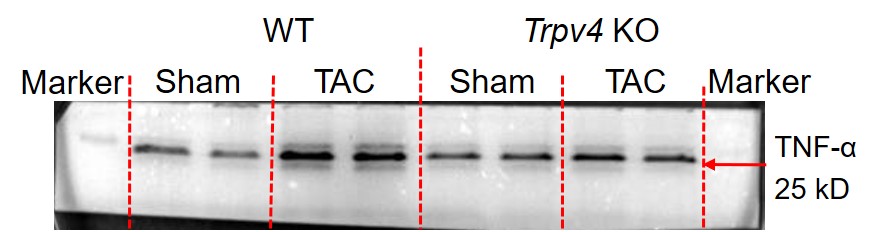

Supplement: Source data 1. [file elife-74519-data1.zip › Source data 1/Western blots/labelled/figure4A-labelled/figure4A-TNF α-3-labelled.jpg]

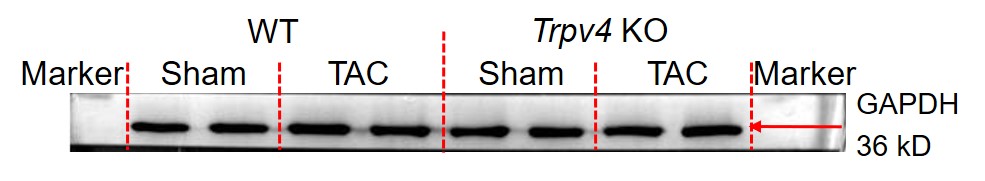

Supplement: Source data 1. [file elife-74519-data1.zip › Source data 1/Western blots/labelled/figure4A-labelled/figure4A-TNF α-GAPDH-1-labelled.jpg]

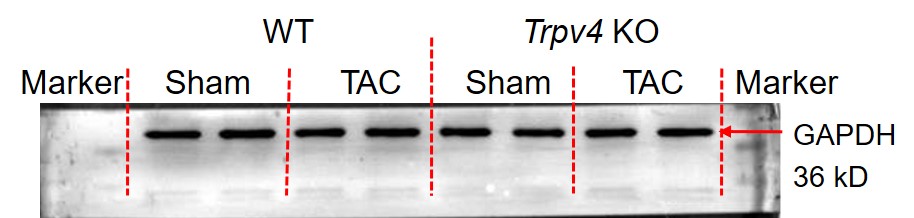

Supplement: Source data 1. [file elife-74519-data1.zip › Source data 1/Western blots/labelled/figure4A-labelled/figure4A-TNF α-GAPDH-2-labelled.jpg]

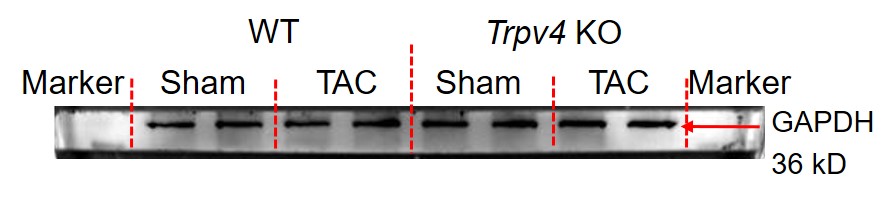

Supplement: Source data 1. [file elife-74519-data1.zip › Source data 1/Western blots/labelled/figure4A-labelled/figure4A-TNF α-GAPDH-3-labelled.jpg]

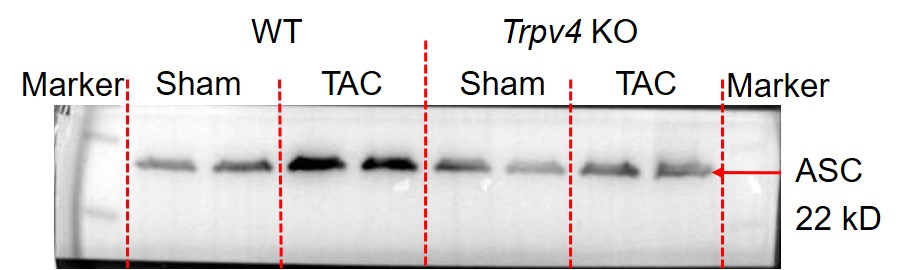

Supplement: Source data 1. [file elife-74519-data1.zip › Source data 1/Western blots/labelled/figure4J-labelled/figure4J-ASC-2-labelled.jpg]

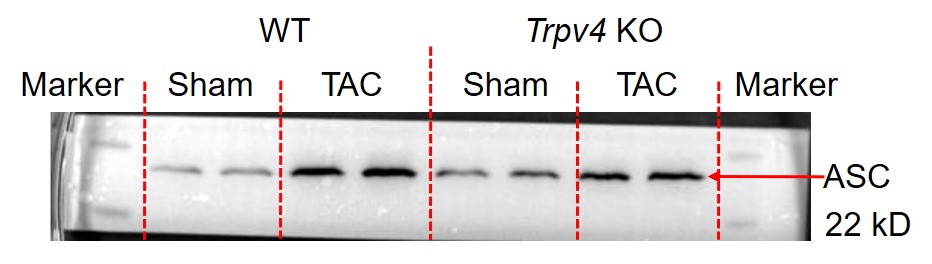

Supplement: Source data 1. [file elife-74519-data1.zip › Source data 1/Western blots/labelled/figure4J-labelled/figure4J-ASC-3-labelled.jpg]

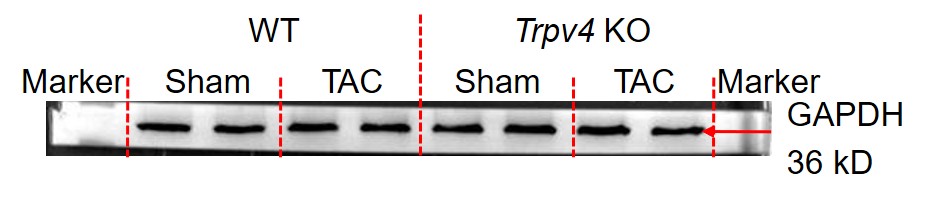

Supplement: Source data 1. [file elife-74519-data1.zip › Source data 1/Western blots/labelled/figure4J-labelled/figure4J-ASC-GAPDH-2-labelled.jpg]

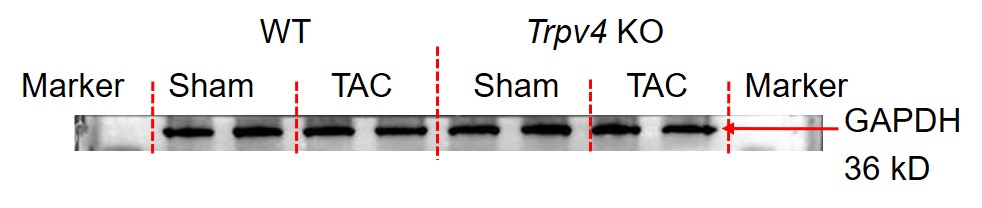

Supplement: Source data 1. [file elife-74519-data1.zip › Source data 1/Western blots/labelled/figure4J-labelled/figure4J-ASC-GAPDH-3-labelled.jpg]

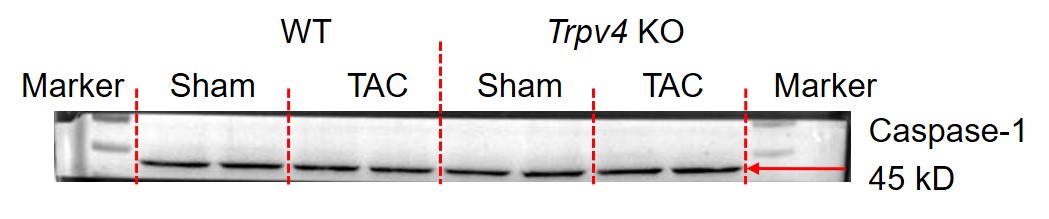

Supplement: Source data 1. [file elife-74519-data1.zip › Source data 1/Western blots/labelled/figure4J-labelled/figure4J-Caspase1-1-labelled.jpg]

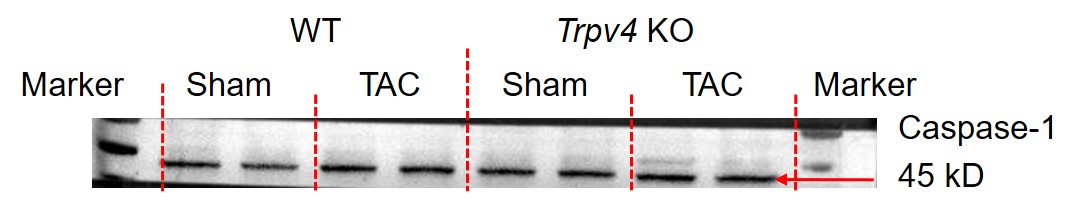

Supplement: Source data 1. [file elife-74519-data1.zip › Source data 1/Western blots/labelled/figure4J-labelled/figure4J-Caspase1-2-labelled.jpg]

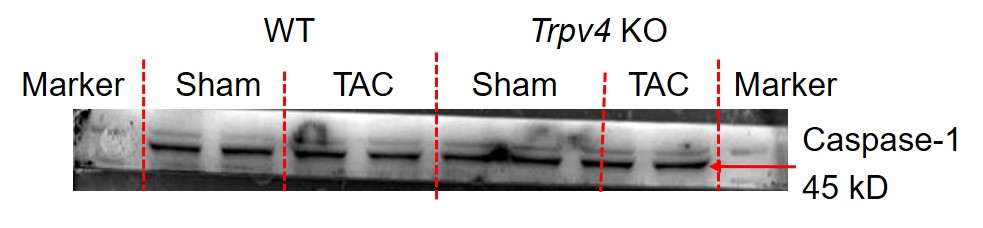

Supplement: Source data 1. [file elife-74519-data1.zip › Source data 1/Western blots/labelled/figure4J-labelled/figure4J-Caspase1-3-labelled.jpg]

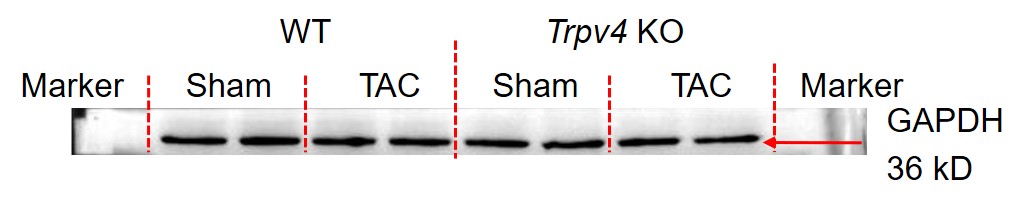

Supplement: Source data 1. [file elife-74519-data1.zip › Source data 1/Western blots/labelled/figure4J-labelled/figure4J-Caspase1-p20-GAPDH-1-labelled.jpg]

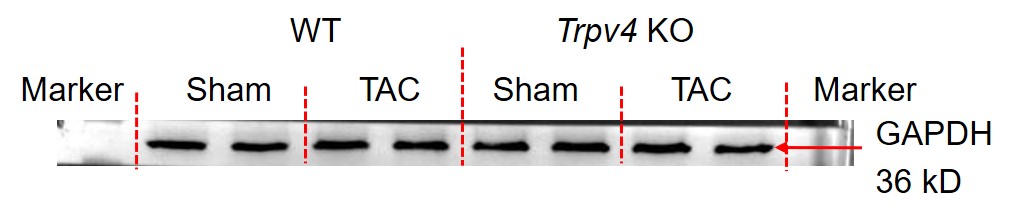

Supplement: Source data 1. [file elife-74519-data1.zip › Source data 1/Western blots/labelled/figure4J-labelled/figure4J-Caspase1-p20-GAPDH-2-labelled.jpg]

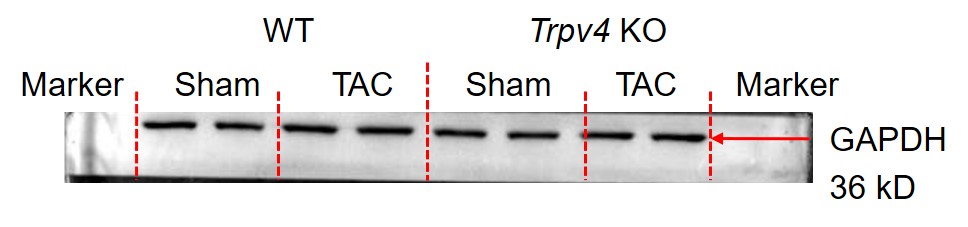

Supplement: Source data 1. [file elife-74519-data1.zip › Source data 1/Western blots/labelled/figure4J-labelled/figure4J-Caspase1-p20-GAPDH-3-labelled.jpg]

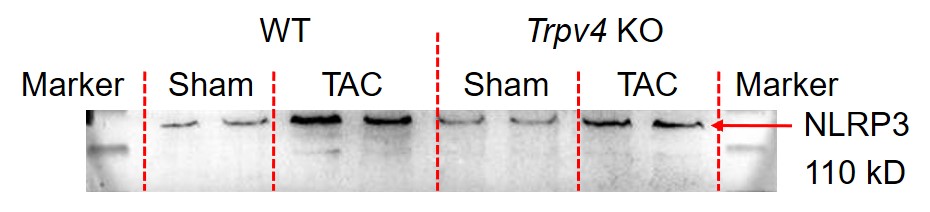

Supplement: Source data 1. [file elife-74519-data1.zip › Source data 1/Western blots/labelled/figure4J-labelled/figure4J-NLRP3-1-labelled.jpg]

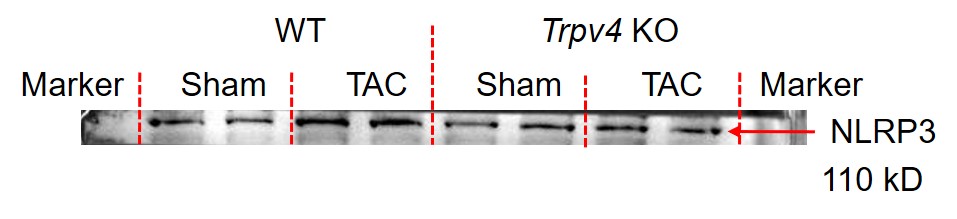

Supplement: Source data 1. [file elife-74519-data1.zip › Source data 1/Western blots/labelled/figure4J-labelled/figure4J-NLRP3-2-labelled.jpg]

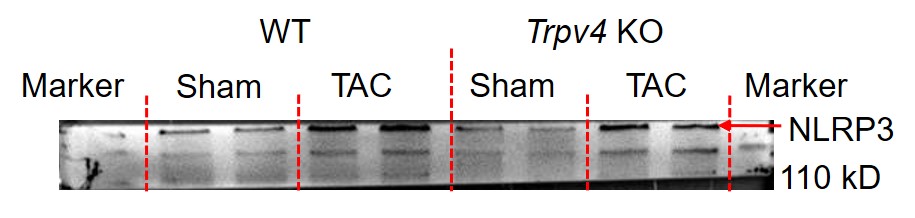

Supplement: Source data 1. [file elife-74519-data1.zip › Source data 1/Western blots/labelled/figure4J-labelled/figure4J-NLRP3-3-labelled.jpg]

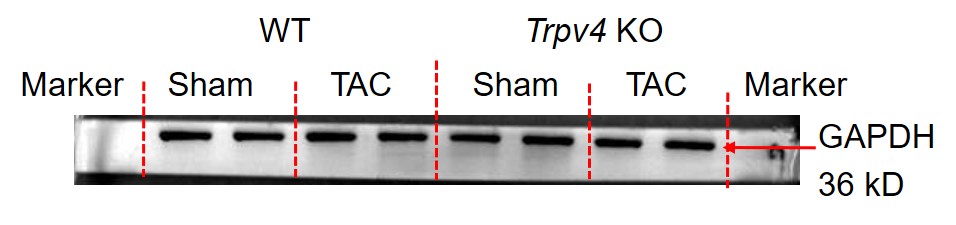

Supplement: Source data 1. [file elife-74519-data1.zip › Source data 1/Western blots/labelled/figure4J-labelled/figure4J-NLRP3-GAPDH-1-labelled.jpg]

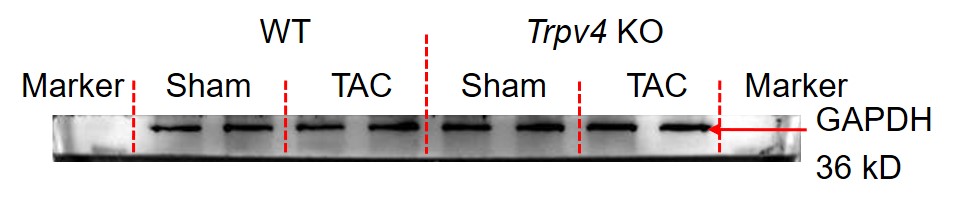

Supplement: Source data 1. [file elife-74519-data1.zip › Source data 1/Western blots/labelled/figure4J-labelled/figure4J-NLRP3-GAPDH-2-labelled.jpg]

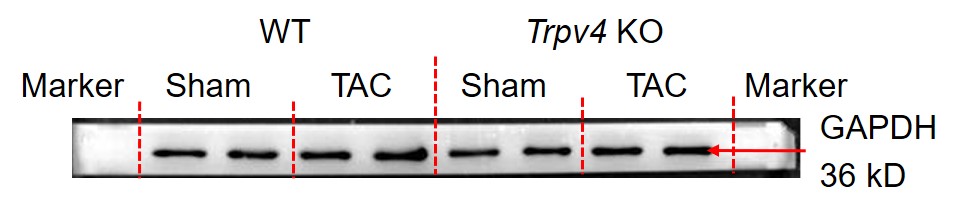

Supplement: Source data 1. [file elife-74519-data1.zip › Source data 1/Western blots/labelled/figure4J-labelled/figure4J-NLRP3-GAPDH-3-labelled.jpg]

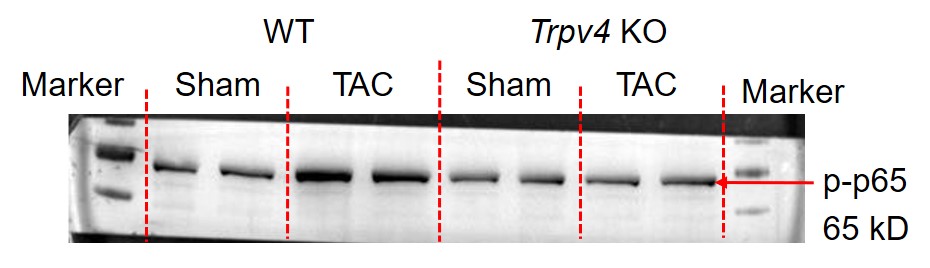

Supplement: Source data 1. [file elife-74519-data1.zip › Source data 1/Western blots/labelled/figure4J-labelled/figure4J-p-p65-1-labelled.jpg]

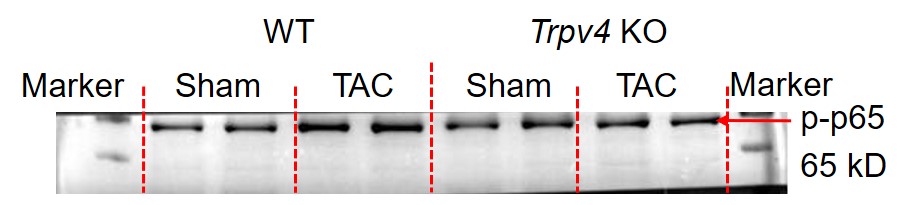

Supplement: Source data 1. [file elife-74519-data1.zip › Source data 1/Western blots/labelled/figure4J-labelled/figure4J-p-p65-2-labelled.jpg]

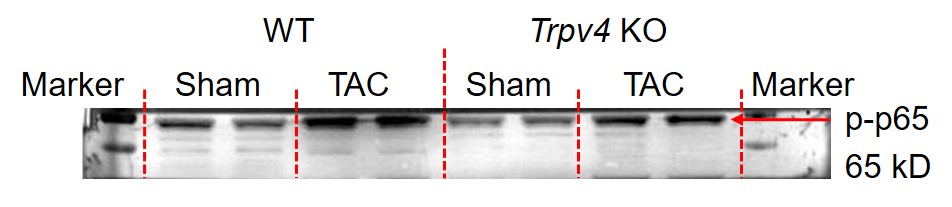

Supplement: Source data 1. [file elife-74519-data1.zip › Source data 1/Western blots/labelled/figure4J-labelled/figure4J-p-p65-3-labelled.jpg]

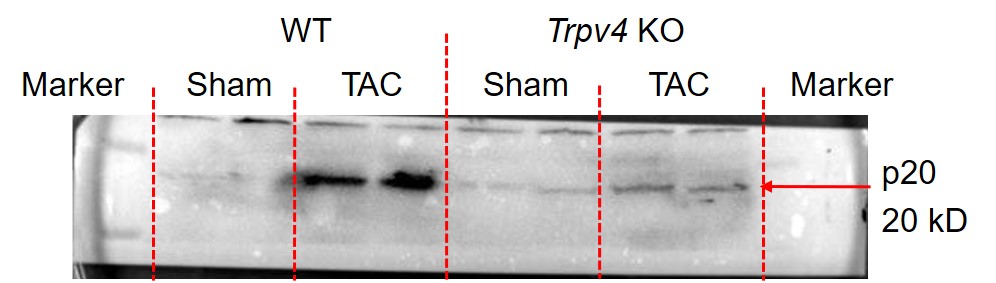

Supplement: Source data 1. [file elife-74519-data1.zip › Source data 1/Western blots/labelled/figure4J-labelled/figure4J-p20-1-labelled.jpg]

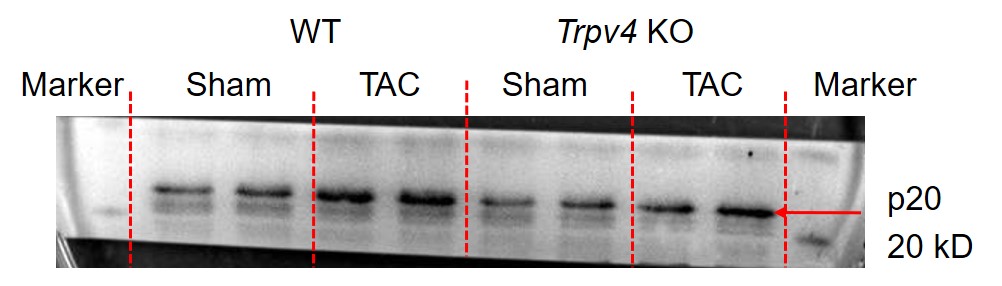

Supplement: Source data 1. [file elife-74519-data1.zip › Source data 1/Western blots/labelled/figure4J-labelled/figure4J-p20-2-labelled.jpg]

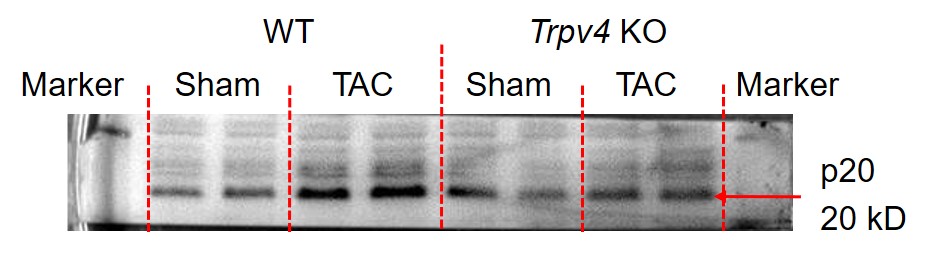

Supplement: Source data 1. [file elife-74519-data1.zip › Source data 1/Western blots/labelled/figure4J-labelled/figure4J-p20-3-labelled.jpg]

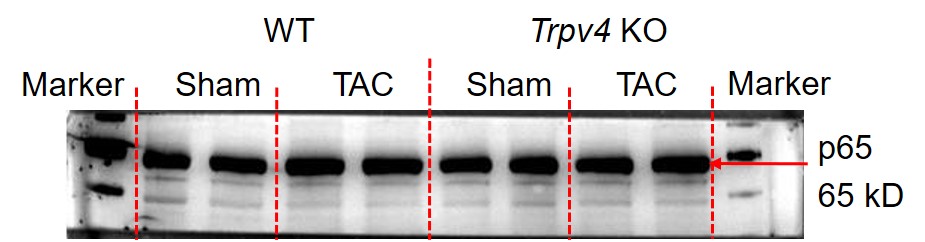

Supplement: Source data 1. [file elife-74519-data1.zip › Source data 1/Western blots/labelled/figure4J-labelled/figure4J-p65-1-labelled.jpg]

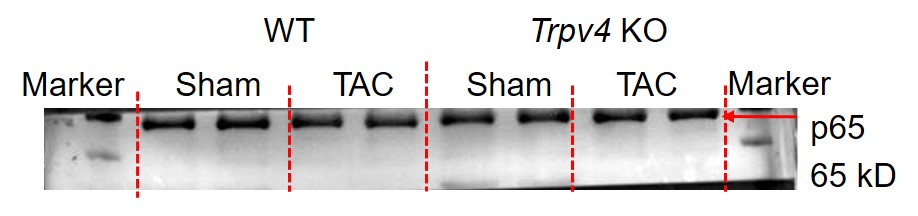

Supplement: Source data 1. [file elife-74519-data1.zip › Source data 1/Western blots/labelled/figure4J-labelled/figure4J-p65-2-labelled.jpg]

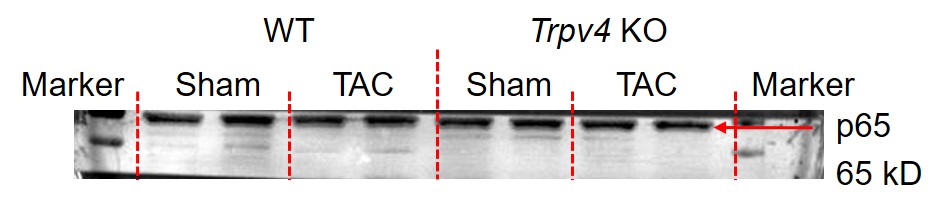

Supplement: Source data 1. [file elife-74519-data1.zip › Source data 1/Western blots/labelled/figure4J-labelled/figure4J-p65-3-labelled.jpg]

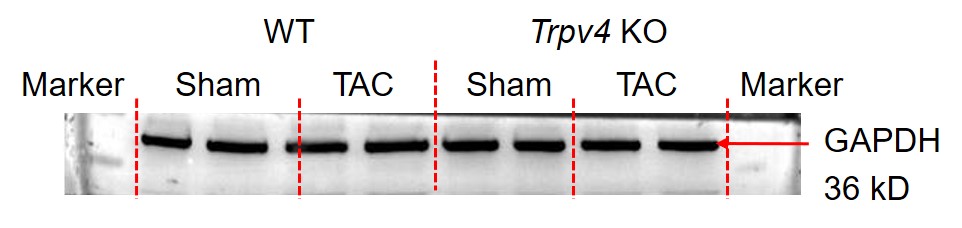

Supplement: Source data 1. [file elife-74519-data1.zip › Source data 1/Western blots/labelled/figure4J-labelled/figure4J-pp65-p65-GAPDH-1-labelled.jpg]

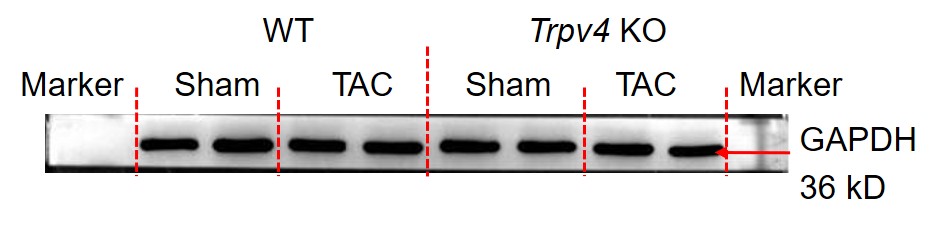

Supplement: Source data 1. [file elife-74519-data1.zip › Source data 1/Western blots/labelled/figure4J-labelled/figure4J-pp65-p65-GAPDH-2-labelled.jpg]

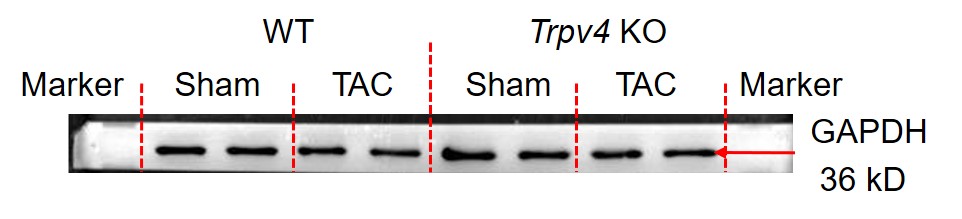

Supplement: Source data 1. [file elife-74519-data1.zip › Source data 1/Western blots/labelled/figure4J-labelled/figure4J-pp65-p65-GAPDH-3-labelled.jpg]

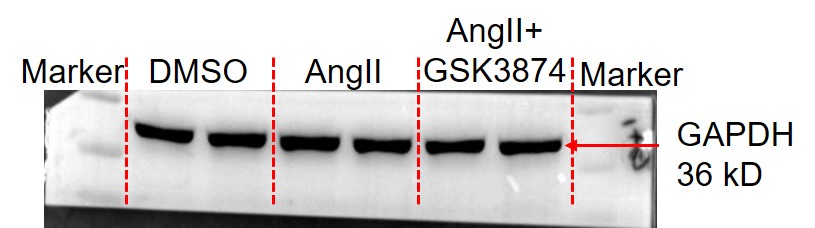

Supplement: Source data 1. [file elife-74519-data1.zip › Source data 1/Western blots/labelled/figure7A-labelled/figure7A-GAPDH-1-labelled.jpg]

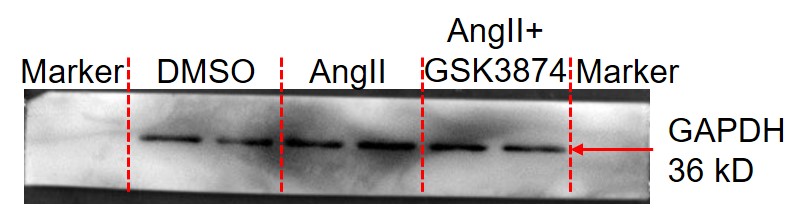

Supplement: Source data 1. [file elife-74519-data1.zip › Source data 1/Western blots/labelled/figure7A-labelled/figure7A-GAPDH-2-labelled.jpg]

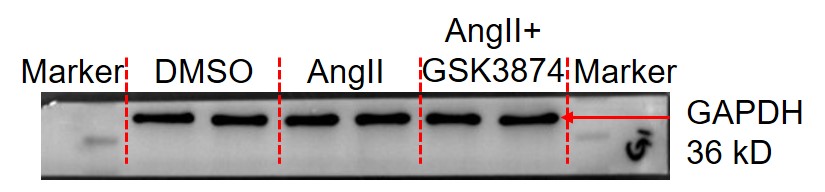

Supplement: Source data 1. [file elife-74519-data1.zip › Source data 1/Western blots/labelled/figure7A-labelled/figure7A-GAPDH-3-labelled.jpg]

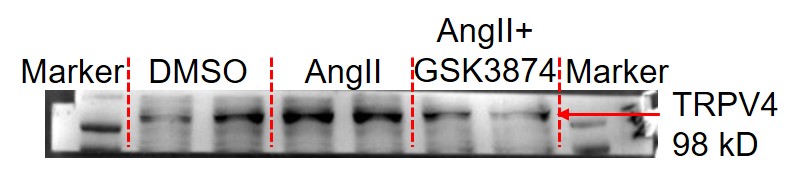

Supplement: Source data 1. [file elife-74519-data1.zip › Source data 1/Western blots/labelled/figure7A-labelled/figure7A-TRPV4-1-labelled.jpg]

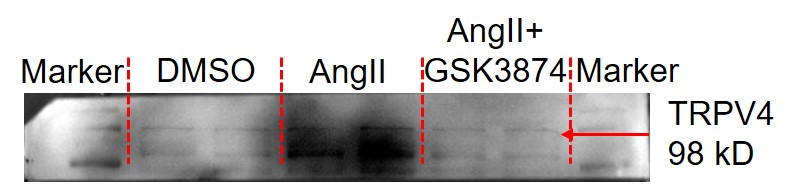

Supplement: Source data 1. [file elife-74519-data1.zip › Source data 1/Western blots/labelled/figure7A-labelled/figure7A-TRPV4-2-labelled.jpg]

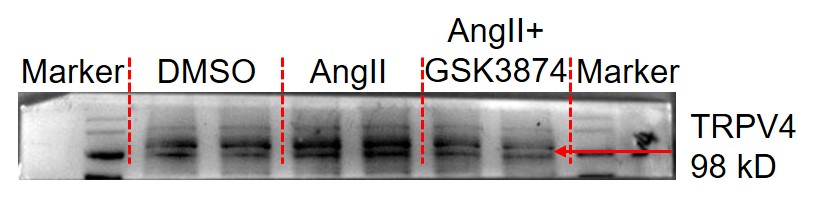

Supplement: Source data 1. [file elife-74519-data1.zip › Source data 1/Western blots/labelled/figure7A-labelled/figure7A-TRPV4-3-labelled.jpg]

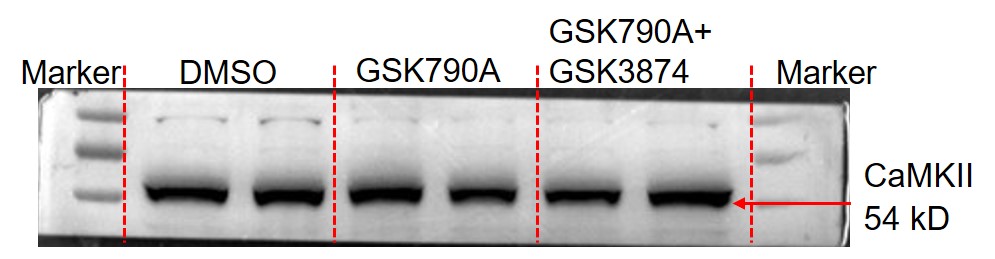

Supplement: Source data 1. [file elife-74519-data1.zip › Source data 1/Western blots/labelled/figure8A-labelled/figure8A-CaMKII-1-labelled.jpg]

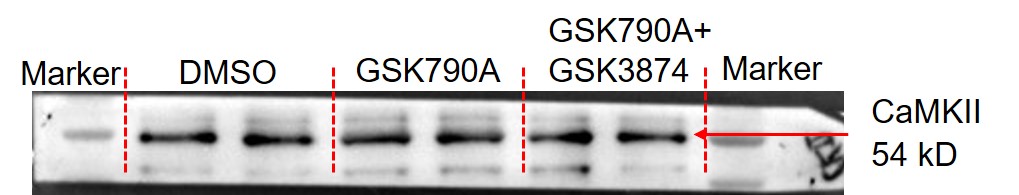

Supplement: Source data 1. [file elife-74519-data1.zip › Source data 1/Western blots/labelled/figure8A-labelled/figure8A-CaMKII-2-labelled.jpg]

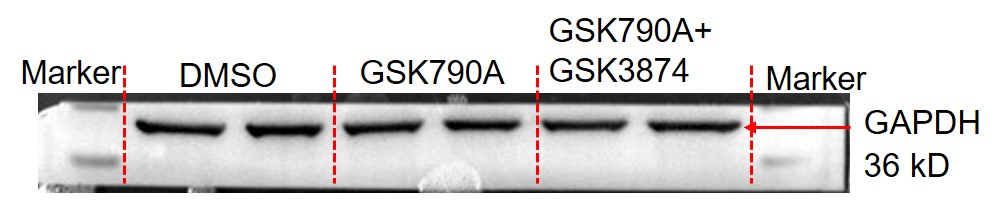

Supplement: Source data 1. [file elife-74519-data1.zip › Source data 1/Western blots/labelled/figure8A-labelled/figure8A-GAPDH-1-labelled.jpg]

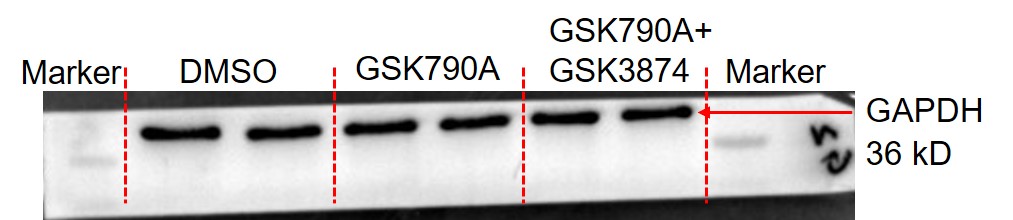

Supplement: Source data 1. [file elife-74519-data1.zip › Source data 1/Western blots/labelled/figure8A-labelled/figure8A-GAPDH-2-labelled.jpg]

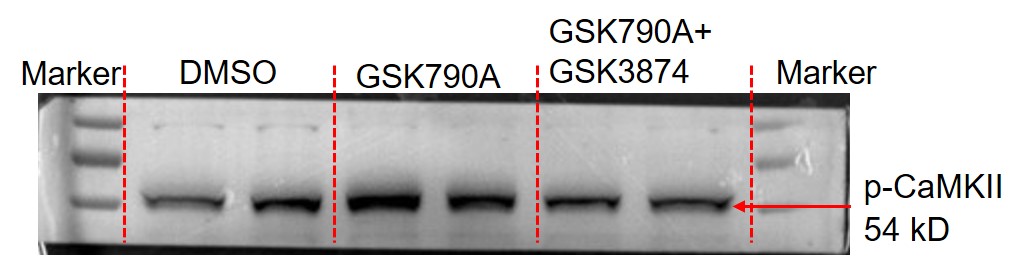

Supplement: Source data 1. [file elife-74519-data1.zip › Source data 1/Western blots/labelled/figure8A-labelled/figure8A-pCaMKII-1-labelled.jpg]

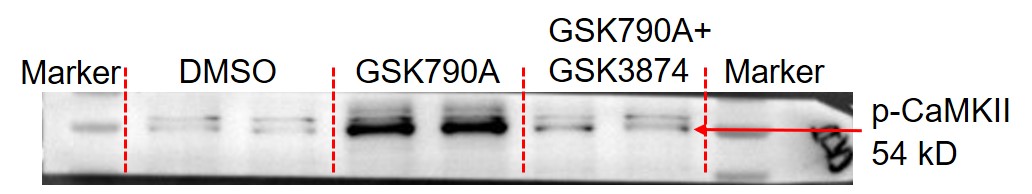

Supplement: Source data 1. [file elife-74519-data1.zip › Source data 1/Western blots/labelled/figure8A-labelled/figure8A-pCaMKII-2-labelled.jpg]

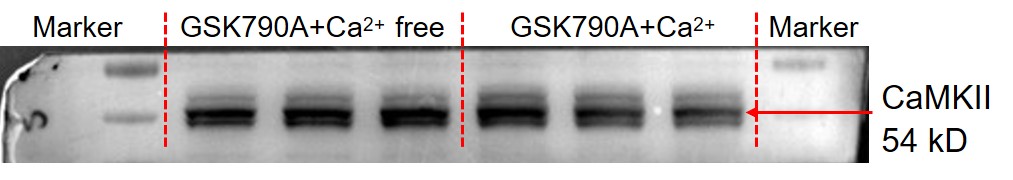

Supplement: Source data 1. [file elife-74519-data1.zip › Source data 1/Western blots/labelled/figure8C-labelled/figure8C-CaMKII-1-labelled.jpg]

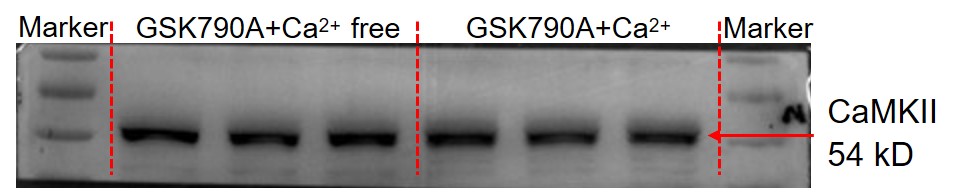

Supplement: Source data 1. [file elife-74519-data1.zip › Source data 1/Western blots/labelled/figure8C-labelled/figure8C-CaMKII-2-labelled.jpg]

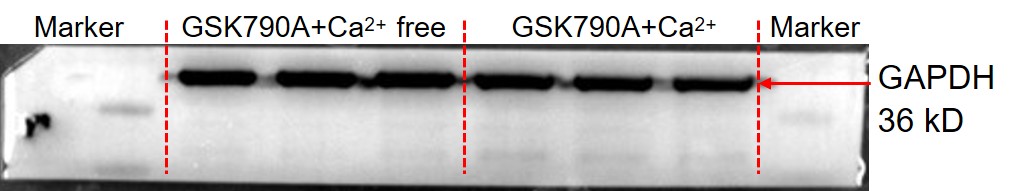

Supplement: Source data 1. [file elife-74519-data1.zip › Source data 1/Western blots/labelled/figure8C-labelled/figure8C-GAPDH-1-labelled.jpg]

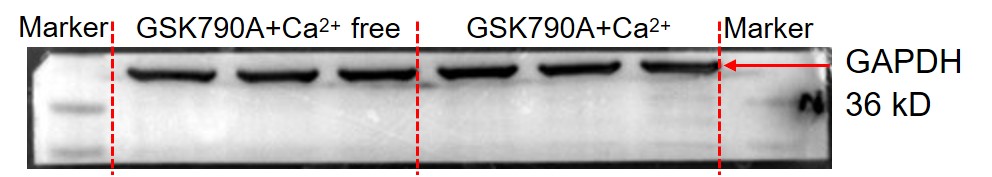

Supplement: Source data 1. [file elife-74519-data1.zip › Source data 1/Western blots/labelled/figure8C-labelled/figure8C-GAPDH-2-labelled.jpg]

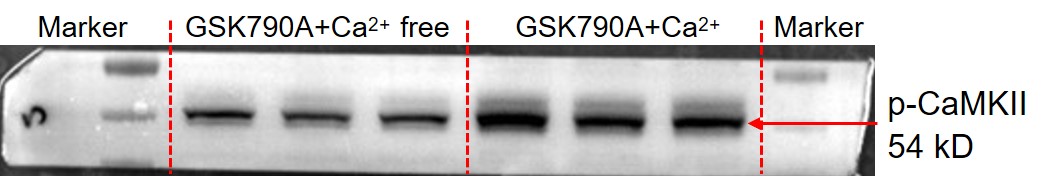

Supplement: Source data 1. [file elife-74519-data1.zip › Source data 1/Western blots/labelled/figure8C-labelled/figure8C-pCaMKII-1-labelled.jpg]

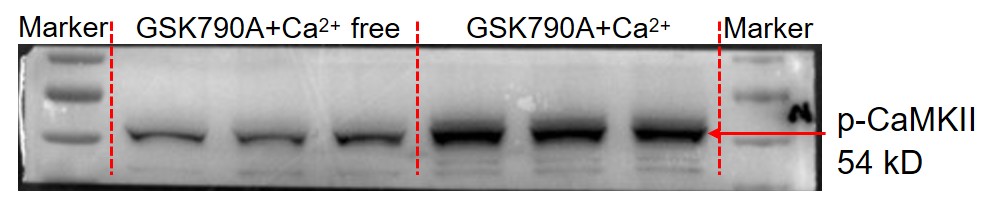

Supplement: Source data 1. [file elife-74519-data1.zip › Source data 1/Western blots/labelled/figure8C-labelled/figure8C-pCaMKII-2-labelled.jpg]

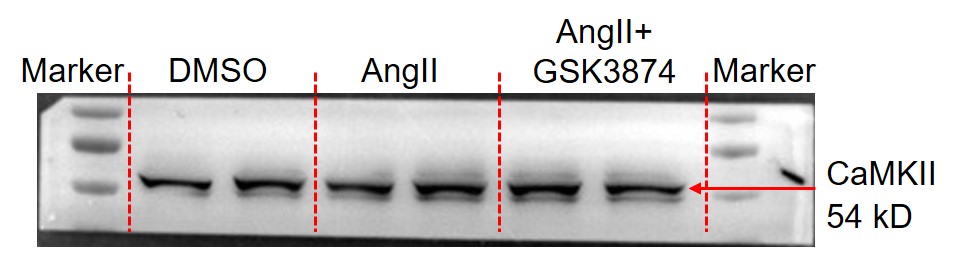

Supplement: Source data 1. [file elife-74519-data1.zip › Source data 1/Western blots/labelled/figure8E-labelled/figure8E-CaMKII-1-labelled.jpg]

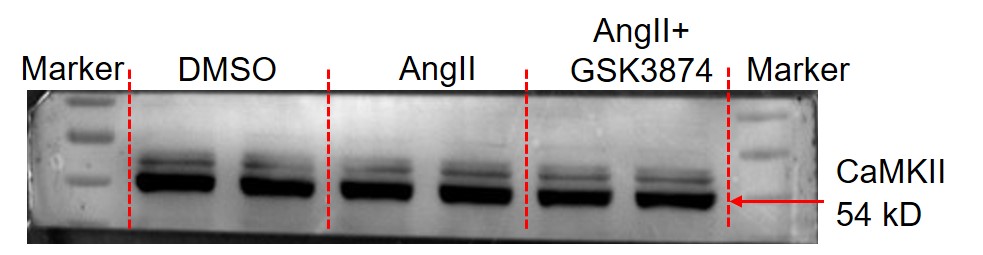

Supplement: Source data 1. [file elife-74519-data1.zip › Source data 1/Western blots/labelled/figure8E-labelled/figure8E-CaMKII-2-labelled.jpg]

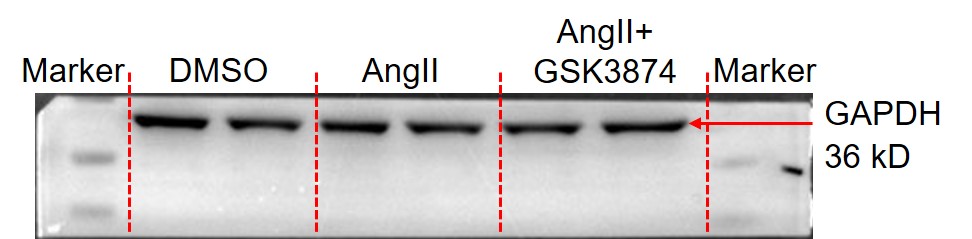

Supplement: Source data 1. [file elife-74519-data1.zip › Source data 1/Western blots/labelled/figure8E-labelled/figure8E-GAPDH-1-labelled.jpg]

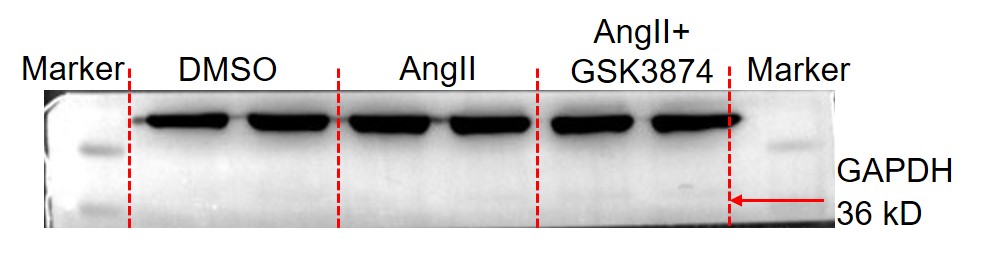

Supplement: Source data 1. [file elife-74519-data1.zip › Source data 1/Western blots/labelled/figure8E-labelled/figure8E-GAPDH-2-labelled.jpg]

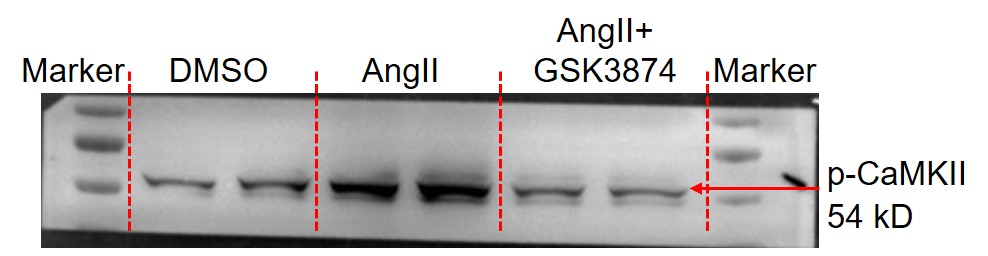

Supplement: Source data 1. [file elife-74519-data1.zip › Source data 1/Western blots/labelled/figure8E-labelled/figure8E-pCaMKII-1-labelled.jpg]

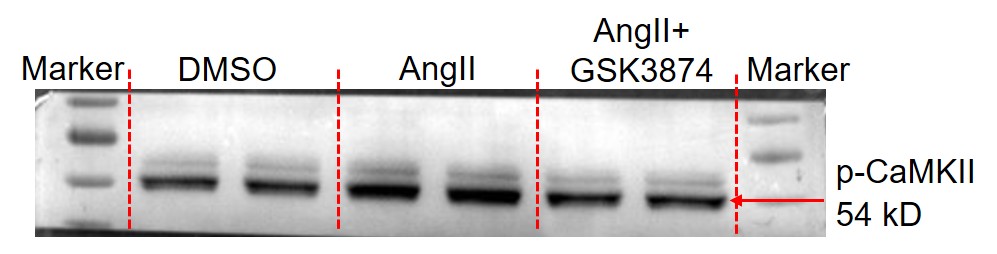

Supplement: Source data 1. [file elife-74519-data1.zip › Source data 1/Western blots/labelled/figure8E-labelled/figure8E-pCaMKII-2-labelled.jpg]

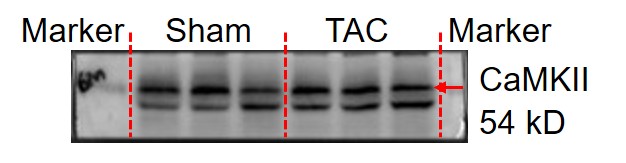

Supplement: Source data 1. [file elife-74519-data1.zip › Source data 1/Western blots/labelled/figure8G-labelled/WT-TAC1w-CaMKII-1-labelled.jpg]

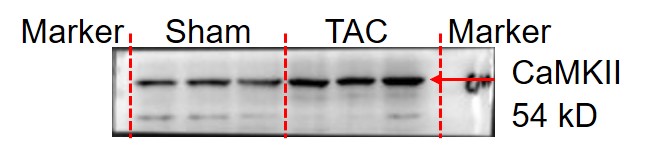

Supplement: Source data 1. [file elife-74519-data1.zip › Source data 1/Western blots/labelled/figure8G-labelled/WT-TAC1w-CaMKII-2-labelled.jpg]

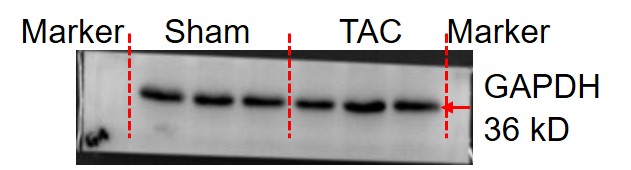

Supplement: Source data 1. [file elife-74519-data1.zip › Source data 1/Western blots/labelled/figure8G-labelled/WT-TAC1w-GAPDH-1-labelled.jpg]

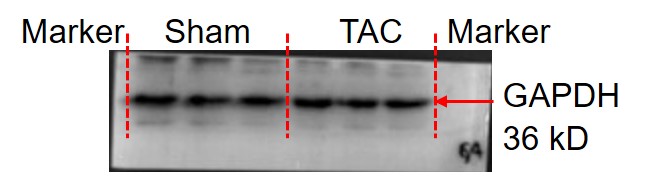

Supplement: Source data 1. [file elife-74519-data1.zip › Source data 1/Western blots/labelled/figure8G-labelled/WT-TAC1w-GAPDH-2-labelled.jpg]

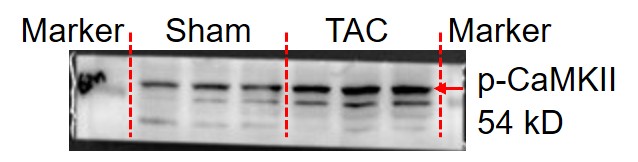

Supplement: Source data 1. [file elife-74519-data1.zip › Source data 1/Western blots/labelled/figure8G-labelled/WT-TAC1w-pCaMKII-1-labelled.jpg]

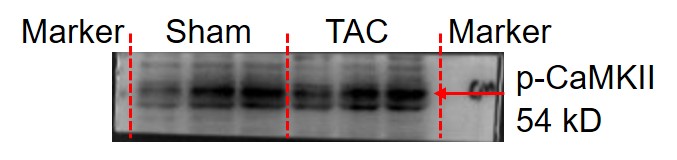

Supplement: Source data 1. [file elife-74519-data1.zip › Source data 1/Western blots/labelled/figure8G-labelled/WT-TAC1w-pCaMKII-2-labelled.jpg]

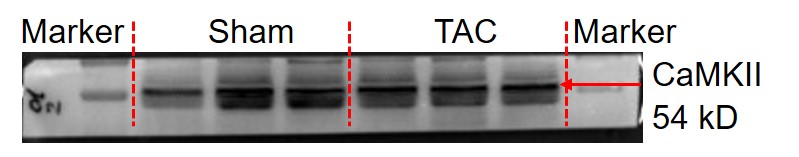

Supplement: Source data 1. [file elife-74519-data1.zip › Source data 1/Western blots/labelled/figure8G-labelled/WT-TAC2d-CaMKII-1-labelled.jpg]

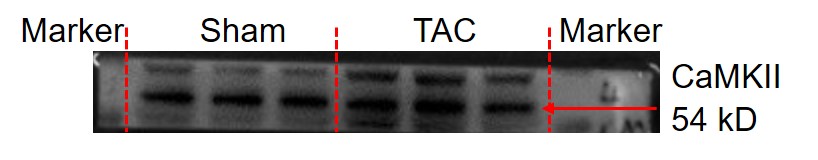

Supplement: Source data 1. [file elife-74519-data1.zip › Source data 1/Western blots/labelled/figure8G-labelled/WT-TAC2d-CaMKII-2-labelled.jpg]

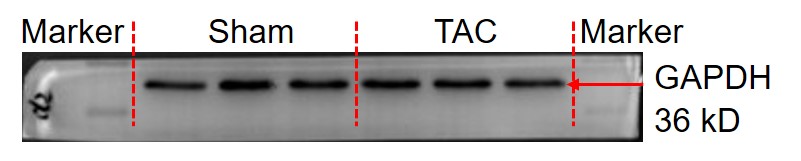

Supplement: Source data 1. [file elife-74519-data1.zip › Source data 1/Western blots/labelled/figure8G-labelled/WT-TAC2d-GAPDH-1-labelled.jpg]

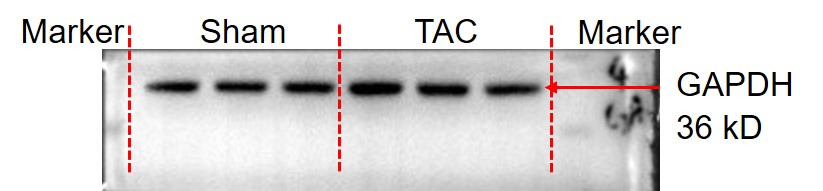

Supplement: Source data 1. [file elife-74519-data1.zip › Source data 1/Western blots/labelled/figure8G-labelled/WT-TAC2d-GAPDH-2-labelled.jpg]

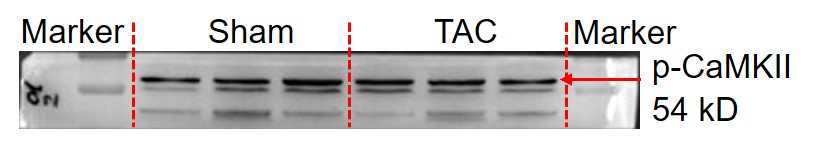

Supplement: Source data 1. [file elife-74519-data1.zip › Source data 1/Western blots/labelled/figure8G-labelled/WT-TAC2d-pCaMKII-1-labelled.jpg]

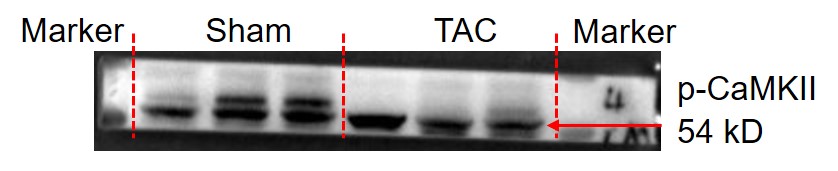

Supplement: Source data 1. [file elife-74519-data1.zip › Source data 1/Western blots/labelled/figure8G-labelled/WT-TAC2d-pCaMKII-2-labelled.jpg]

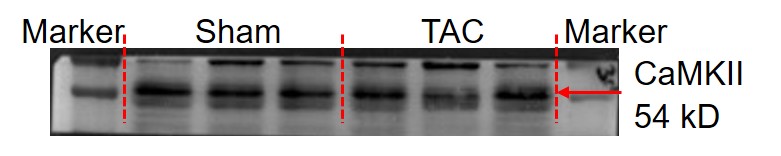

Supplement: Source data 1. [file elife-74519-data1.zip › Source data 1/Western blots/labelled/figure8G-labelled/WT-TAC2w-CaMKII-1-labelled.jpg]
